# Trithorax regulates long-term memory in *Drosophila* through epigenetic maintenance of mushroom body metabolic state and translation capacity

**DOI:** 10.1371/journal.pbio.3003004

**Published:** 2025-01-27

**Authors:** Nicholas Raun, Spencer G. Jones, Olivia Kerr, Crystal Keung, Emily F. Butler, Kumari Alka, Jonathan D. Krupski, Robert A. Reid-Taylor, Veyan Ibrahim, MacKayla Williams, Deniz Top, Jamie M. Kramer

**Affiliations:** 1 Department of Biochemistry and Molecular Biology, Dalhousie University, Halifax, Canada; 2 Department of Physiology and Pharmacology, University of Western Ontario, London, Canada; 3 Department of Cell Biology, University of Alberta, Edmonton, Canada; Stony Brook University Medical Center: Stony Brook University Hospital, UNITED STATES OF AMERICA

## Abstract

The role of epigenetics and chromatin in the maintenance of postmitotic neuronal cell identities is not well understood. Here, we show that the histone methyltransferase Trithorax (Trx) is required in postmitotic memory neurons of the *Drosophila* mushroom body (MB) to enable their capacity for long-term memory (LTM), but not short-term memory (STM). Using MB-specific RNA-, ChIP-, and ATAC-sequencing, we find that Trx maintains homeostatic expression of several non-canonical MB-enriched transcripts, including the orphan nuclear receptor *Hr51*, and the metabolic enzyme *lactate dehydrogenase* (*Ldh)*. Through these key targets, Trx facilitates a metabolic state characterized by high lactate levels in MBγ neurons. This metabolic state supports a high capacity for protein translation, a process that is essential for LTM, but not STM. These data suggest that Trx, a classic regulator of cell type specification during development, has additional functions in maintaining underappreciated aspects of postmitotic neuron identity, such as metabolic state. Our work supports a body of evidence suggesting that a high capacity for energy metabolism is an essential cell identity characteristic for neurons that mediate LTM.

## Abstract 2

Epigenetic mechanisms govern cell fate decisions during cell division but what is their role in long-lived post-mitotic neurons? This study shows that the Trithorax histone methyltransferase regulates the metabolic state of mushroom body neurons and is necessary for long term memory formation in Drosophila.

## Introduction

Brain function depends on the coordinated activities of many different cell types. The cell identity of postmitotic neurons is maintained by different combinations of transcription factors (TFs), termed terminal selectors, which activate the expression of effector gene networks [1,2]. Effector genes define critical neuron identity features such as neurotransmitter type and synaptic surface receptors. Terminal selectors that maintain postmitotic neuron identity have been elucidated in the simple and well-defined nervous system of *Caenorhabditis elegans* [3–6] and in more complex nervous systems of *Drosophila melanogaster* and mice [7–12]. While some terminal selectors are required to maintain an array of general cell identity features [13,14], others have more specific roles in the activation of specific aspects of neuronal identity [15,16]. For example, the TF Dimmed (Dimm) is required specifically to maintain the expression of genes required for secretory function in *Drosophila* secretory neurons [15,17,18]. We still lack a complete understanding of the factors that maintain different aspects of neuron identity in a complex nervous system. In addition, the different types of cell identity features that are important for specialized neural functions are not fully elucidated.

Epigenetic modification of chromatin structure has been proposed as a mechanism through which cells establish and maintain their cell identity [1,19]. The Trithorax group proteins are among the most well-studied epigenetic regulators. Trithorax group proteins counteract Polycomb group proteins to activate the expression of homeotic (hox) genes, which define body segment identity during development [20]. While the role of Trithorax and Polycomb group proteins in developmental cell type specification is well defined, their function in maintaining postmitotic cell identity is not extensively explored.

The namesake of the Trithorax group proteins, encoded by the *Drosophila trithorax (trx)* gene, is a histone methyltransferase that methylates histone H3 on lysine 4 (H3K4), a chromatin mark associated with active gene expression. In *Drosophila*, there are 3 enzymes that catalyze H3K4 methylation, Trx, Trithorax related (Trr) and Set1 [21]. While Trr and Set1 regulate bulk levels of H3K4 mono- and tri-methylation, respectively, Trx seems to have a more selective role in modification of specific loci [21,22]. In humans, heterozygous mutations in the orthologous H3K4 methyltransferases (Trx = KMT2A/B, Trr = KMT2C/D, Set1 = SETD1A/B) cause neurodevelopmental disorders (NDDs), such as autism spectrum disorder and intellectual disability [23–28]. Individuals with NDDs related to mutations in H3K4 methyltransferases do not generally have morphological brain abnormalities [24,25,27–30], suggesting that their cognitive deficits are caused by dysfunction of postmitotic adult neurons. In this study, we investigated the function of Trx in adult postmitotic memory neurons of the *Drosophila* mushroom body (MB). Our data reveals a role for Trx in homeostatic epigenetic maintenance of MB metabolic state, which is emerging as an essential aspect of postmitotic cell identity for neurons that mediate LTM [31–34].

## Results

### Trx is required in adult MB neurons for LTM, but not STM

**Fig 1:**
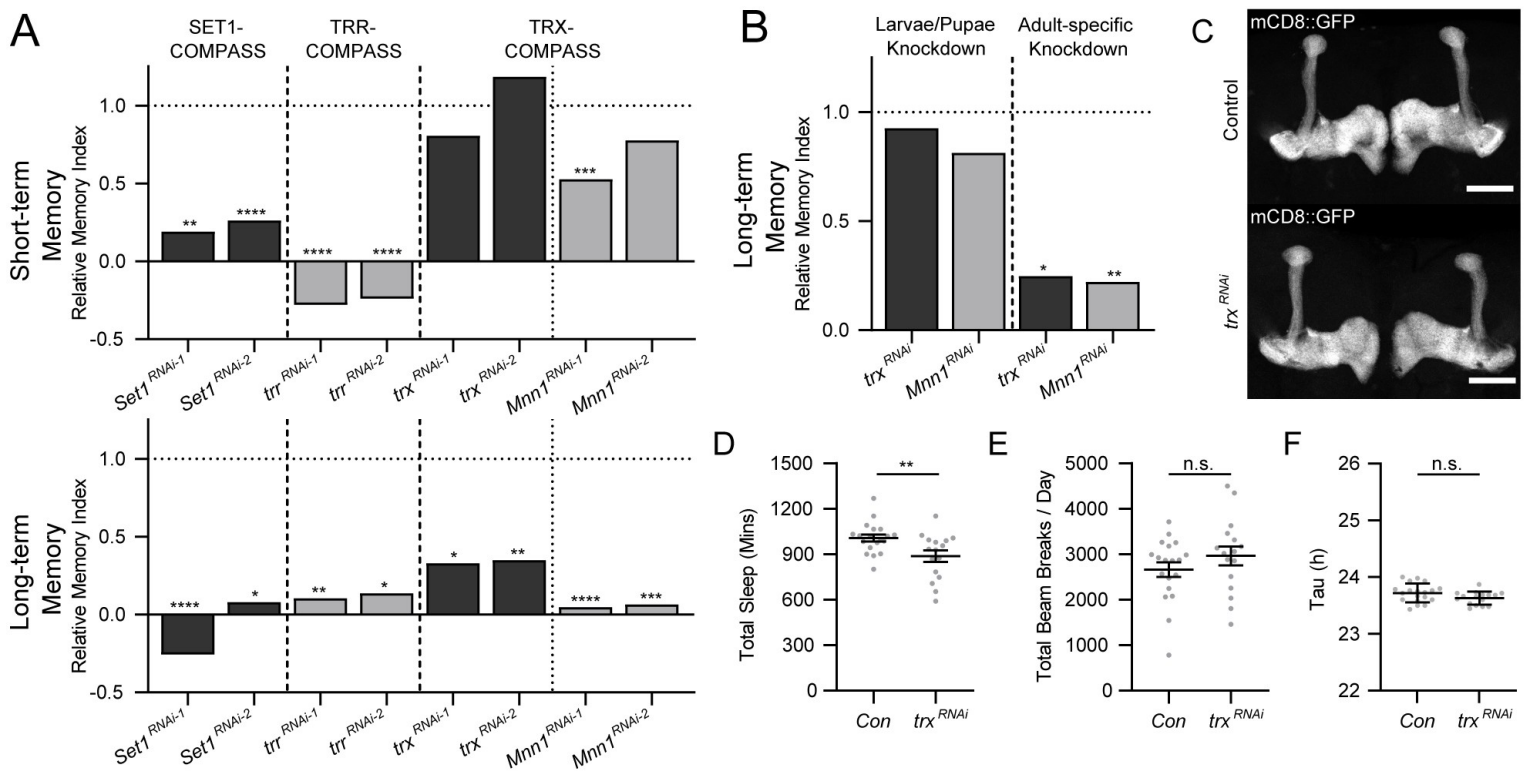
Trx is required in the MB for long-term courtship memory. ** (A)** Bar graphs showing relative MIs (average MI knockdown/average MI control) for courtship STM (upper panel) and LTM (lower panel) after knockdown of *Drosophila* H3K4 methyltransferases in the MB. Two RNAi lines were used for each gene: *Set1*, *trr*, *trx*, and *Mnn1*. The type of COMPASS complex they are associated with is indicated above. Previously published data for Set1 [27] is shown for comparison. The raw data and full genotypes are shown in **S1A Fig, S5 Table, and S1 Data**. **(B)** Bar graphs showing relative MIs for courtship LTM when *trx* and *Mnn1* MB knockdown was limited to larvae and pupae stages (left panel) or adult (right panel) using Gal80^ts^. The raw data, full genotypes, and temperature shift protocols are shown in **S1B Fig, S5 Table, and S1 Data**. Statistical significance in **(A)** and **(B)** was determined using a randomization test with 10,000 bootstrap replicates, comparing RNAi knockdown strains to genetic background controls containing *Gal4/Gal80*^*ts*^, with no *UAS-RNAi* (indicated by horizontal dashed lines). **(C)** Confocal Z-stack projections showing the MB morphology of *trx*^*RNAi*^ flies (*UAS-mCD8*::*GFP/{trx-RNAi-2}v60000;R14H06-Gal4/UAS-Dcr2*) compared to controls (*UAS-mCD8*::*GFP/v60000; R14H06-Gal4/UAS-Dcr2*), visualized by expression of *UAS-mCD8*::*GFP* with *R14H06-Gal4*. Scale bar indicates 50 microns. **(D–F)** Dot plots showing **(D)** sleep, **(E)** activity, and **(F)** circadian rhythm of *trx*^*RNAi*^ flies (*UAS-Dcr2/{trx-RNAi-2}v60000;R14H06-Gal4/+*) compared to controls (*UAS-Dcr2/v60000;R14H06-Gal4/+*). Statistical significance was determined using Student’s *t* test. Raw data for **D and E** is available in **S1 Data**. *n*.*s*. not significant, *p < 0.05, **p < 0.01, ***p < 0.001, ****p < 0.0001. LTM, long-term memory; MB, mushroom body; MI, memory index; STM, short-term memory.

We investigated the function of the 3 known *Drosophila* H3K4 methyltransferases—Set1, Trr, and Trx—in the well-characterized memory neurons of the *Drosophila* MB [35]. The MB is required for short- and long-term memory (STM and LTM) in flies, but not for learning [36–39]. The post mitotic MB-specific *R14H06-Gal4* driver [27,40–42] was used to drive expression of 2 unique *UAS-RNAi* lines for each *Drosophila* H3K4 methyltransferase. As controls, we tested genetically matched flies that contain the Gal4 transgene and no *UAS-RNAi*, or a non-targeting *UAS-mCherry-RNAi* (see Methods for technical details). MB-specific RNAi flies and genetic background controls were tested for STM and LTM using the classic *Drosophila* memory assay, courtship conditioning [39,43,44]. Our previous work showed that Set1 is required in the MB for courtship STM and LTM [27] and that Trr is required for STM [45]. Here, we show that MB-specific *trr*^*RNAi*^ also reduces LTM (**Figs 1A and S1A and S1 Data**), suggesting a broad role for both Trr and Set1 in regulating both STM and LTM in postmitotic MB neurons. In contrast, MB-specific *trx*^*RNAi*^ only reduced LTM ability, not STM (**Figs 1A and S1A and S1 Data**), suggesting a more specific role in the MB for Trx.

Each *Drosophila* H3K4 methyltransferase acts in a unique conformation of the complex of proteins associated with Set1 (COMPASS) [21]. The COMPASS complexes consist of 4 common subunits and additional complex-specific subunits [21]. Trx acts in the TRX-COMPASS complex where it interacts with the specific co-factor Menin 1 (Mnn1) [46–48]. MB-specific *Mnn1*^*RNAi*^ also induced LTM defects, with 2 independent RNAi lines causing complete loss of LTM ability compared to controls (**Figs 1A and S1A and S1 Data**). In contrast, only one of 2 *Mnn1*^*RNAi*^ lines caused a 50% reduction in STM ability (**Figs 1A and S1A and S1 Data**). Overall, this data suggests that the TRX-COMPASS complex has a role in maintaining the capacity of MB neurons for LTM, but not STM.

To understand if LTM loss resulted from a developmental or adult-specific role of Trx and Mnn1, we limited the postmitotic MB knockdown to either the larval/pupal stage, or the adult stage, using temperature sensitive Gal80 (Gal80^ts^) [49]. Gal80 inhibits Gal4 at 18°C but is deactivated at 29°C allowing for temporal activation of *UAS-RNAi* transgenes. When *trx*^*RNAi*^ and *Mnn1*^*RNA*i^ were induced at 29°C during the larval and pupal stages and silenced at 18°C in adults, we found that memory was unaffected compared to genetic background controls (**Figs 1B and S1B and S1 Data**). However, when RNAi expression was silenced in larvae and pupae at 18°C and activated at 29°C immediately after eclosion in adults, LTM was lost in *trx*^*RNAi*^ and *Mnn1*^*RNAi*^ flies (**Figs 1B and S1B and S1 Data**). Controls containing *R14H06-Gal4*, *Gal80*^*ts*^, and *UAS-Dcr-2* but no *UAS-RNAi* transgene, or a *UAS-RNAi* transgene with no *Gal4* or *Gal80*^*ts*^ showed normal memory, demonstrating that LTM loss was not due to the temperature shift alone (**Figs 1B and S1B and S1 Data**). In addition, gross MB morphology appeared normal in MB specific *trx*^*RNAi*^ flies even when Gal80^ts^ was not used to limit knockdown of Trx to adult flies (**Fig 1C**). We also tested the role of Trx in other adult behaviors that are linked to the MB, including sleep and activity [50]. MB-specific *Trx*^*RNAi*^ flies showed a small but significant decrease in total sleep compared to controls (**Fig 1D and S1 Data**), with all sleep differences occurring during the dark period of the day-night cycle (**S1C Fig and S1 Data**). However, total activity (**Fig 1E and S1 Data**) and rhythmicity of sleep (**Fig 1F and S1 Data**) were normal. Taken together, these data demonstrate that Trx and its specific cofactor Mnn1 support the capacity of adult postmitotic MB neurons to form LTM, without having a major effect on development, or other MB functions.

### Trx is dispensable for memory induced gene transcription

**Fig 2:**
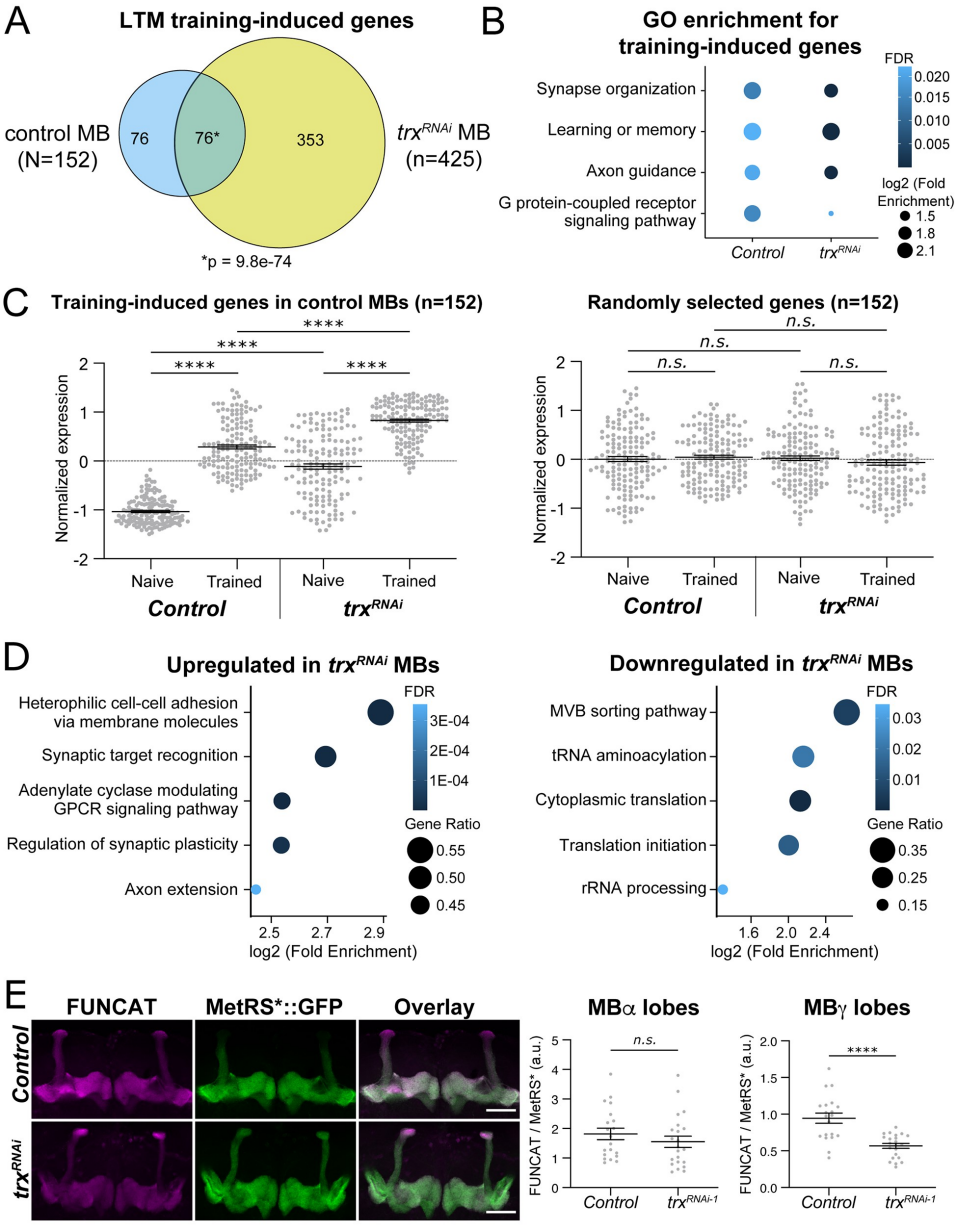
Trx regulates translation genes and protein synthesis in MB neurons. **(A)** Venn diagram showing the overlap between LTM TIGs identified in *trx*^*RNAi*^ MBs (*UAS-unc84*::*GFP/{trx-RNAi-2}v60000;R14H06-Gal4/UAS-Dcr2*) and genetic background control MBs (*UAS-unc84*::*GFP/v60000;R14H06-Gal4/UAS-Dcr2*). The *p*-value was determined using a hypergeometric test. **(B)** Bubble plot summarizing the gene ontology enrichment analysis of MB-specific LTM TIGs in *trx*^*RNAi*^ and control genotypes. The most enriched representative terms are shown. Dot color indicates FDR, and size indicates log2(fold-change). Statistical significance was determined using Fisher’s exact test. **(C)** Dot plots showing normalized expression values for 152 LTM TIGs identified in control MBs (left panel) and 152 randomly selected genes (right panel). *P*-values were determined using pairwise Wilcoxon tests. **(D)** Bubble plots summarizing gene ontology enrichment analysis of genes that are up-regulated (left panel) and down-regulated (right panel) in *trx*^*RNAi*^ MBs compared to control MBs. The 5 most enriched and representative terms are shown. Dot color indicates FDR, and size indicates the ratio of genes found in our gene list compared to the reference. *P*-values were determined using Fisher’s exact test. **(E)** Representative confocal slices showing translation levels in *trx*^*RNAi*^ MBs (UAS-Dcr2/{trx-RNAi-2}v60000);R14H06-Gal4/UAS-metRS*::*GFP*) compared to genetic controls (UAS-Dcr2/v60000;R14H06-Gal4/UAS-metRS*::*GFP*), as measured by FUNCAT. Left panel—FUNCAT signal, middle panel—MetRS*::GFP, right panel—overlay. Scale bars indicate 50 microns. Quantification of FUNCAT signal (FUNCAT/MetRS*::GFP) is shown for the MBα (left) and MBγ (right) lobes. Raw data are available in **S1 Data**. *P*-values were calculated using Student’s *t* test. ****p < 0.*0001*. LTM, long-term memory; MB, mushroom body; TIG, training-induced gene.

A key difference between LTM and STM is that LTM requires de novo gene transcription and protein translation, whereas STM does not [51,52]. Considering the known role of Trx as a transcriptional activator, we hypothesized that it might be involved in LTM training-induced gene (TIG) expression in the MB. We therefore used isolation of nuclei in a tagged cell type (INTACT) followed by RNA-sequencing [40,53] to analyze the MB transcriptome in *trx*^*RNAi*^ MBs compared to controls, before (naïve) and after (trained) LTM training (see Methods for details). In genetic background control MBs, we observed 152 TIGs that were induced 1 h after LTM training compared to naive flies. Surprisingly, in *trx*^*RNAi*^ MBs, we detected a much greater number of TIGs, *n* = 425 (**Fig 2A**). TIGs identified in *trx*^*RNAi*^ MBs and the controls were enriched for GO annotations related to memory formation, such as synapse organization (GO:0050808), learning or memory (GO:0007611), and G protein-coupled receptor signaling pathway (GO:0007185) (**Fig 2B**). As a group, the 152 TIGs identified in control MBs have significantly higher expression in both naïve and trained states in *trx*^*RNAi*^ MBs (**Fig 2C**). This difference is not due to a broad difference in gene expression in *trx*^*RNAi*^ MBs, since the expression of 152 randomly chosen genes show identical expression levels in control and *trx*^*RNAi*^ MBs, regardless of training status (**Fig 2C**). Overall, 76 genes were found to be significantly induced by training in both in control and *trx*^*RNAi*^ MBs, an overlap that is significantly more than expected by random chance (*p* = 9.8e-74, hypergeometric test) (**Fig 2A**). There were also 76 TIGs that were significantly induced in controls, but not in *trx*^*RNAi*^ MBs. However, these genes show elevated naive expression levels in *trx*^*RNAi*^ MBs, comparable to the level observed in trained controls (**S2A Fig**). This suggests that many TIGs are induced prior to training in *trx*^*RNAi*^ MBs. In summary, MB-specific *trx*^*RNAi*^ results in a greater number of TIGs, and generally higher expression of TIGs in both naïve and trained conditions compared to controls (**Figs 2D and S2A**). Overall, we find no evidence that Trx is important in the MB for LTM TIG activation. Our data suggests the opposite, that LTM TIG expression is overactive when Trx is depleted from the MB. This was unexpected due to the known role of Trx in depositing H3K4 methylation associated with activation of gene expression.

### Trx promotes translation in the MB

Since Trx does not appear to activate LTM TIG transcription, we examined other processes that might be compromised in *trx*^*RNAi*^ MBs. Comparing RNA-seq data from control and *trx*^*RNAi*^ MBs (**S1 Table**), we found that loss of Trx resulted in significant up-regulation of synaptic genes (**Fig 2D**). This is consistent with the observation that LTM TIGs are overactive in *trx*^*RNAi*^ MBs (**Figs 2C and S2A**). In contrast, down-regulated genes in *trx*^*RNAi*^ MBs were enriched for processes related to translation (**Fig 2D**). Of the 861 down-regulated genes in *trx*^*RNAi*^ MBs, 68 are involved in translation (GO:0006412), including key genes involved in translation initiation (GO:0003743, e.g., eIF2β and 4 eIF2B subunits), 26 structural constituents of the ribosome (GO:0003735), and 11 tRNA synthetase genes (GO:0043039).

Since translation is required for LTM and not STM, it is possible that the LTM defect associated with loss of Trx in the MB is caused by defective MB translation capacity. We therefore used fluorescent non-canonical amino acid tagging (FUNCAT) [54,55] to quantitatively evaluate translation in the MB. To perform FUNCAT in the MB, a UAS responsive mutant methionyl-tRNA synthetase (UAS-MetRS*::*GFP*) was expressed using *R14H06-Gal4*. Expression of MetRS*::GFP allows for the incorporation of the non-canonical amino acid azidonorleucine (ANL) into nascent proteins [55]. ANL is not available in the normal fly media and is not incorporated by wild-type MetRS, so it is only incorporated into proteins in tissues where MetRS*::GFP is expressed after flies are fed ANL-supplemented food. Proteins containing ANL can then be fluorescently labeled using click chemistry [56,57] and visualized by confocal microscopy. FUNCAT analysis in the MB revealed that *trx*^*RNAi*^ MBs have significantly lower ANL labeling than controls in the MBγ lobes, but not the MBα lobes (**Fig 2E and S1 Data**), providing direct in vivo evidence that some regions of *trx*^*RNAi*^ MBs are deficient in translation. Differences in FUNCAT signal were not likely due to differences in ANL dietary intake, since MB-specific *trx*^*RNAi*^ flies showed no changes in feeding behavior compared to controls (**S2B Fig and S1 Data**). Taken together, these results suggest that *trx*^*RNAi*^ MBs have reduced translation capacity in the MBγ lobes, which is likely underlying the observed LTM-specific memory defect (**Fig 1A**). The reduced translation capacity of Trx depleted MBγ neurons might also underly the observed transcriptional overactivation of LTM TIGs (**Figs 2C and S2A**), as a compensatory mechanism. Interestingly, the MBγ neurons are the main MB cell type implicated in courtship memory [58–60].

### Identification of direct Trx target genes in the MB

**Fig 3:**
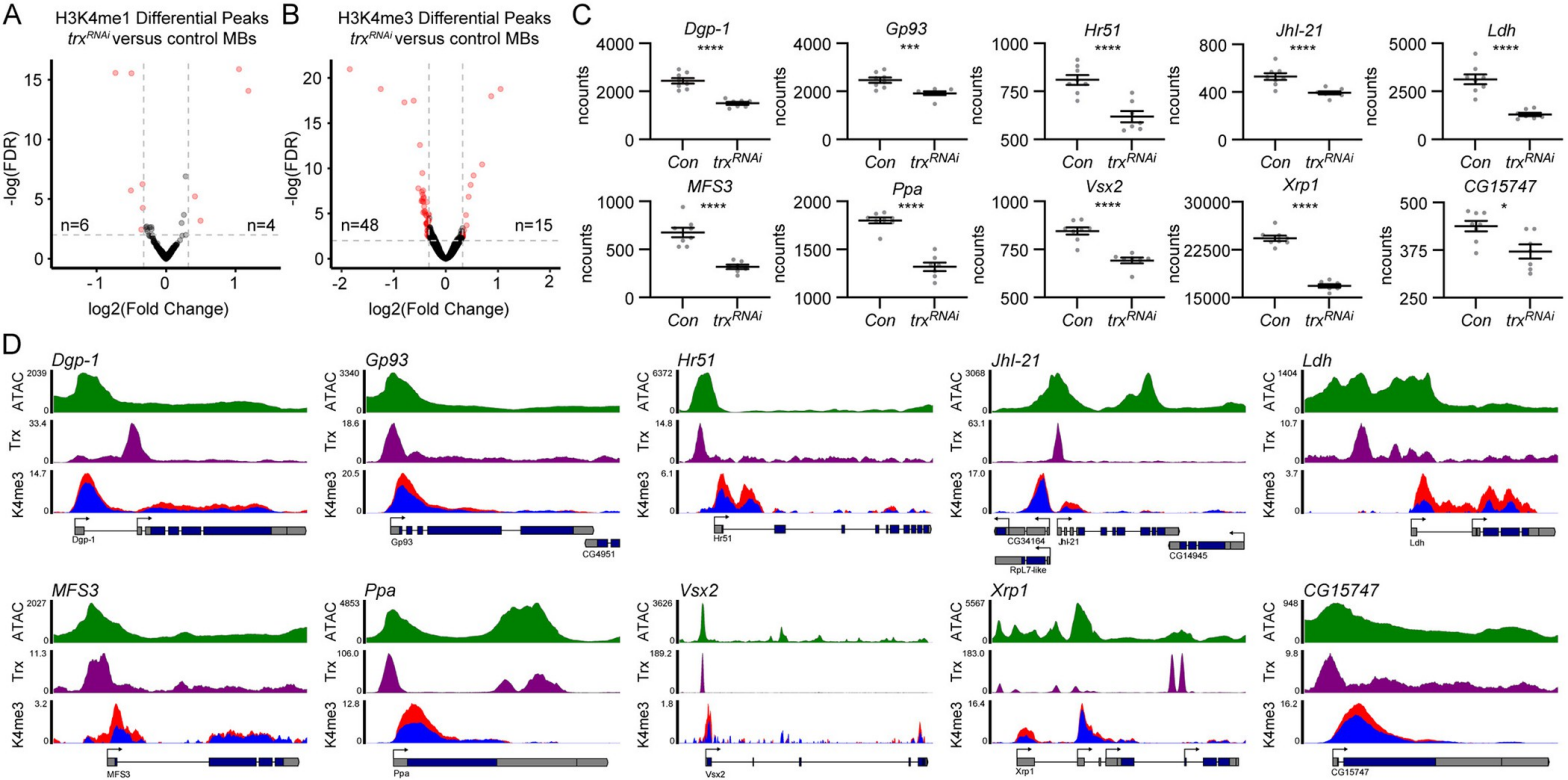
Identification of direct Trx target genes in the MB. **(A, B)** Volcano plots showing differential **(A)** H3K4me1 and **(B)** H3K4me3 peaks identified by comparing *trx*^*RNAi*^ MBs (*UAS-unc84*::*GFP/{trx-RNAi-2}v60000;R14H06-Gal4/UAS-Dcr2*) to genetic background control MBs (*UAS-unc84*::*GFP/v60000;R14H06-Gal4/UAS-Dcr2*). Red dots indicate significant peaks with an FDR < 0.01 and Fold change > 1.25, indicated by dashed lines. Statistical significance was determined using a Wald test. **(C)** Dot plots showing the normalized counts (ncounts) from RNA-seq for 10 genes that were found to be significantly down-regulated in *trx*^*RNAi*^ MBs compared to genetic background control MBs. Statistical significance was determined using a Wald test. **(D)** Genome browser tracks showing chromatin accessibility (ATAC-seq–top panel), Trx DNA binding sites (ChIP-seq–middle panel), and H3K4me3 (ChIP-seq–bottom panel) for 10 Trx target genes. H3K4me3 tracks display values from both the control (red) and *trx*^*RNAi*^ (blue) MBs. Gene models and transcripts are indicated below, with alternative start sites indicated by arrows. *p < 0.05, ***p < 0.001, ****p < 0.0001. MB, mushroom body.

We next sought to understand how Trx promotes translation through its function as a H3K4 methyltransferase. We performed ChIP-seq using H3K4me1 and H3K4me3 antibodies on INTACT-isolated MB nuclei from *trx*^*RNAi*^ and control flies. Potential target H3K4 methylation sites in the MB genome were identified by looking for reduction, or loss, of H3K4 methylation peaks following Trx depletion. We observed 6 significantly reduced H3K4me1 peaks (**Fig 3A and S2 Table**) and 48 reduced H3K4me3 peaks (**Fig 3B and S3 Table**) (FDR < 0.05, Fold change > 1.25) in *trx*^*RNAi*^ MBs, compared to controls. To determine if Trx has the potential to bind at or near these sites in the genome, we used available published Trx ChIP-seq data from S2 cells [61]. Since genomic binding sites can vary in different cell types, we overlapped S2-cell Trx binding sites with MB-specific regions of open chromatin that we identified using Assay for Transposase Accessible Chromatin followed by next generation sequencing (ATAC-seq, see Methods). In 2 independent replicates of INTACT isolated MB nuclei, we identified highly consistent ATAC-seq peaks (**S3 Fig**) representing regions of open chromatin in the MB genome. Open chromatin is highly predictive for tissue-specific transcription factor activity [62–64]; therefore, the presence of experimentally determined S2 cell Trx binding sites in regions of MB open chromatin, suggests that Trx would likely bind at these sites in MB cells. Of the 48 genes with reduced H3K4me3 peaks, 22 were associated with Trx binding sites in MB open chromatin (**Fig 3D)** and 10 of these (*MFS3*, *Hr51*, *Dgp-1*, *Ldh*, *JhI-21*, *Ppa*, *Xrp1*, *Gp93*, *Vsx2*, and *CG15747*) also had reduced mRNA levels in *trx*^*RNAi*^ MBs compared to controls (**Fig 3C**). These data identify a group of genes that are likely activated by Trx directly through its role as an H3K4 methyltransferase in the MB.

### Trx direct target genes encompass novel MB-enriched genes that are required for LTM

**Fig 4:**
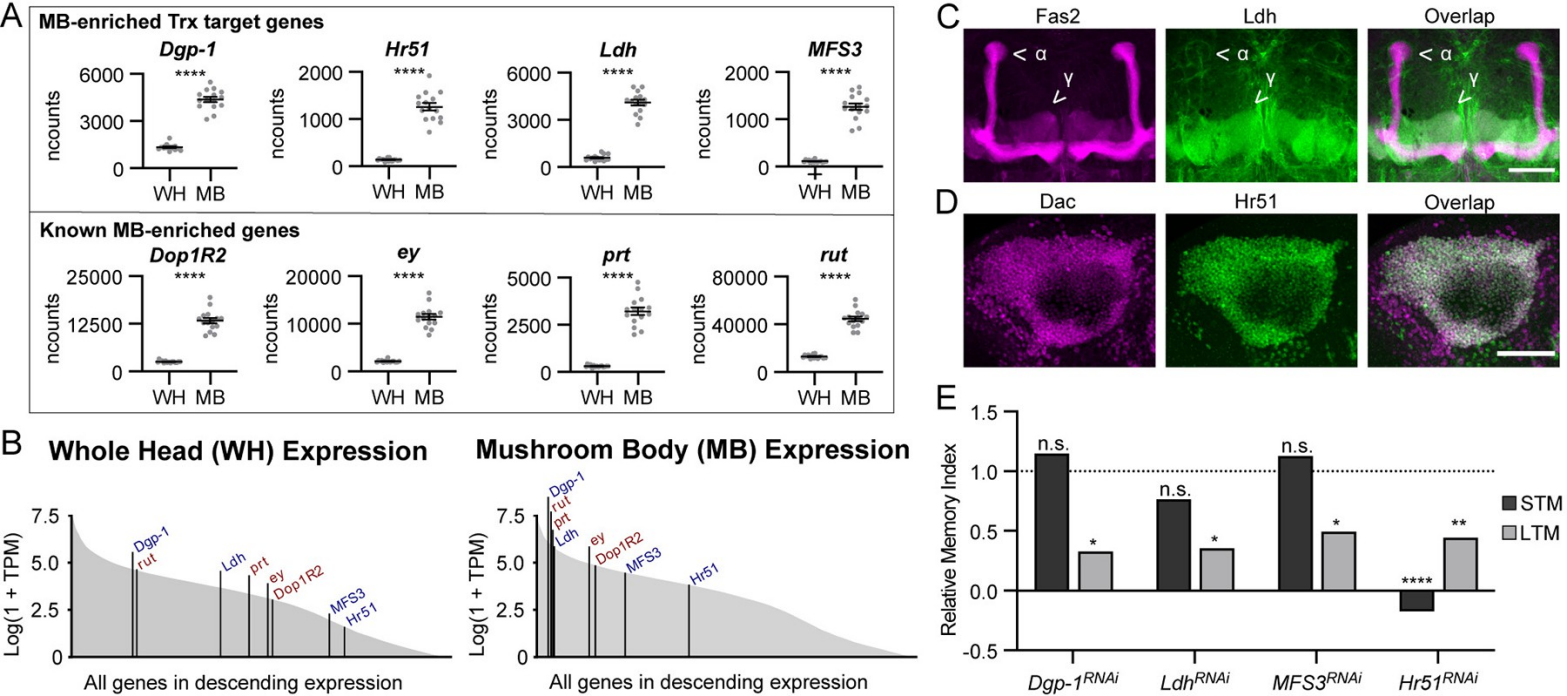
Trx target genes are enriched in the MB and required for MB function. **(A)** Dot plots showing the RNA-seq normalized counts (ncounts) compared between mRNA isolated from the WH and from MB. The upper panel indicates Trx target genes identified here, and the lower panel shows 4 previously known MB-enriched transcripts. Genotype = *UAS-unc84*::*GFP/+;R14H06-Gal4/+*. *P*-values were calculated using a Wald test. **(B)** Area plots showing the log of 1+ TPM for all genes expressed in the WH (left panel) or MB (right panel) in descending order. The rank of each Trx target gene is marked and labelled in blue, and the known MB enriched genes in red. (**C, D**) Confocal Z-stack projections showing **(C)** MB lobes labeled with anti-Fas2 (left panel) and Ldh::GFP (middle panel), and **(D)** MB nuclei labelled with anti-Dac (left panel) and Hr51::GFP (middle panel). Scale bars indicate 50 microns. **(E)** Bar graphs showing the relative MI (average MI knockdown/average MI control) for courtship STM (dark gray) and LTM (light gray) upon MB-specific *Dgp-1*^*RNAi*^, *Ldh*^*RNAi*^, *MFS3*^*RNAi*^, and *Hr51*^*RNAi*^. For *Hr51*, RNAi knockdown was limited to adult flies using Gal80^ts^. *Dgp-1*, *Ldh*, and *MFS3* knockdowns were performed without Gal80^ts^. The relative MI of controls containing *Gal4 and/or Gal80*^*ts*^ but no *UAS-RNAi* is indicated by a horizontal dotted line. Raw data and full genotypes are shown in **S4E Fig, S5 Table, and S1 Data**. Statistical significance between control and RNAi knockdown MI was determined using a randomization test with 10,000 bootstrap replicates. *n*.*s*. not significant, *p < 0.05, **p < 0.01, ***p < 0.001, ****p < 0.0001. LTM, long-term memory; MB, mushroom body; MI, memory index; STM, short-term memory; TPM, transcripts per million; WH, whole head.

Considering the classic known role of Trx in cell and tissue specification [65], we examined whether Trx target genes are relevant for MB neuron identity. To this end, we generated RNA-seq data from MB-specific INTACT and compared it to whole head (WH) nuclear RNA-seq data, as we have done previously [40]. Six of the 10 identified Trx target genes showed significantly enriched expression in the MB compared to WH (**S4 Table**). Four of these, *Dgp-1*, *Hr51*, *Ldh*, and *MFS3*, had greater than 2-fold enrichment of mRNA levels in the MB compared to the WH, similar to what we observe for several known MB-enriched genes that are commonly used as markers to define MB neurons in single-cell RNA-sequencing analysis, including; *ey*, *rut*, *Dop1R2*, and *prt* (**Fig 4A**) [66,67]. Both the known and the newly identified MB-enriched transcripts showed varied levels of expression in WH but were among the top 50% of expressed genes in the MB (**Fig 4B**). *Dgp-1* and *Ldh* are among the highest expressed MB enriched genes, similar to the established MB-enriched genes, *rut* and *prt*. *Ldh* and *MFS3* have among the largest increase in expression from WH to MB relative to other MB-enriched genes (**Fig 4B**).

To validate MB enrichment of the Trx target genes, we generated or obtained transgenic flies expressing tagged proteins under the control of their endogenous gene regulatory elements for Hr51 (BDSC #38650), Ldh [68], MFS3 [69], and Dgp-1 (see Methods). MFS3 and Dgp-1 were both clearly present in the MB but did not show obvious enrichment compared to the surrounding brain tissue (**S4A and S4B Fig**). In contrast, Hr51 and Ldh proteins both showed striking enrichment in the adult MB (**Figs 4C, 4D, S4C and S4D**). Interestingly, Ldh protein showed enriched localization to the MBγ neurons and not the MBα/β and α’/β’ neurons (**Fig 4C**). Ldh enrichment in the MBγ neurons is clear, despite also being expressed in some other neurons and glia throughout the adult brain (**Figs 4C and S4C**). Notably, the MBγ lobe is the part of the MB that is known to underly courtship memory and was also the region of the MB where translation was compromised in *trx*^*RNAi*^ MBs (**Fig 2E**) [39,58]. Ldh has not previously been identified as an MB-enriched protein, but was indicated as a candidate MB-enriched gene by single-cell RNA-seq [66].

Hr51 was clearly localized in Dac-positive nuclei of Kenyon cells, with limited expression in the rest of the brain (**Figs 4D and S4D**). This expression pattern is consistent with previous observations showing nuclear localization of Hr51 in MB neurons [70]. While Hr51 is known to have an important role in MB development and is linked to larval MB neuron identity [71,72], its expression and role in the adult MB has not previously been described. These data suggest that Trx is required to facilitate high MB expression levels of a selected subset of novel MB-enriched transcripts, including *Ldh*, which is specifically limited to the MBγ neurons.

To assess the functional relevance of these MB-enriched Trx target genes, we tested their role in courtship memory. MB-specific *Dgp-1*^*RNAi*^, *Ldh*^*RNAi*^, and *MFS3*^*RNAi*^ reduced LTM, but not STM (**Figs 4E and S4E and S1 Data**). This phenotype was confirmed with a second non-overlapping RNAi line for *Ldh* (**S4E Fig and S1 Data**). Hr51 was previously reported to have a role in MBγ neuron remodeling during pupal metamorphosis [72]. Accordingly, MB-specific *Hr51*^*RNAi*^ resulted in complete absence of the MBγ lobe (**S4F Fig**). When expression of *Hr51*^*RNAi*^ was limited to the adult fly using Gal80^ts^, we still observed loss of STM and LTM compared to controls (**Figs 4E and S4E and S1 Data**). Notably, MB morphology was normal with adult-specific knockdown of Hr51, as expected, since Hr51 controls MB remodeling during the pupal stage of development (**S4F Fig**). The role of Hr51 in MB metamorphosis, STM, and LTM suggests that it may have a broader role in the MB compared to Trx and its other MB-enriched target transcripts, *Dgp-1*, *Ldh*, and *MFS3*, which are only required for LTM, and do not regulate MB morphogenesis.

We next asked whether Trx MB target genes are redundantly targeted by the other *Drosophila* H3K4 methyltransferases, Set1 or Trr. To this end, we performed MB-specific INTACT followed by RNA-seq in *Set1*^*RNAi*^ and *trr*^*RNAi*^ flies, which are deficient in STM and LTM (**Fig 1A**) [27,45]. We compared genes that were significantly down-regulated in *Set1*^*RNAi*^, *trr*^*RNAi*^, and *trx*^*RNAi*^ MBs to genetic background control MBs. While there was a significant level of overlap between genes regulated by Set1, Trr, and Trx, most down-regulated genes were unique to one knockdown condition (**S4G Fig**). MB-specific *trx*^*RNAi*^ resulted in the greatest proportion of unique down-regulated genes, 78%, compared to *trr*^*RNAi*^, at 63%, and *Set1*^*RNAi*^, at 51% (**S4G Fig**). The overlap of down-regulated genes observed between *trr*^*RNAi*^ and *Set1*^*RNAi*^ MBs was statistically more enriched than the overlap with genes down-regulated in *trx*^*RNAi*^ MBs (**S4H Fig**). Importantly, none of the MB-enriched Trx target genes identified here (**Fig 4**) show reduced expression in *Set1*^*RNAi*^ or *trr*^*RNAi*^ MBs (**S4I and S4J Fig**). Therefore, while there is likely a high level of redundancy in the target genes of H3K4 methyltransferases in the MB, the Trx targets identified here represent a group of genes for which Trr and Set1 cannot provide sufficient compensation to achieve the needed homeostatic expression level.

Taken together, we have identified a group of MB-enriched transcripts that are activated by Trx and required in the MB for normal LTM. These novel MB-enriched Trx target genes are distinct from the known developmental hox target genes and expressed at a level similar to several established MB-enriched genes that are commonly used to define MB cell types. This suggests that Trx targets have a critical role in maintaining the capacity of MB neurons to mediate LTM.

### Trx target genes affect the translation capacity of MB memory neurons

**Fig 5:**
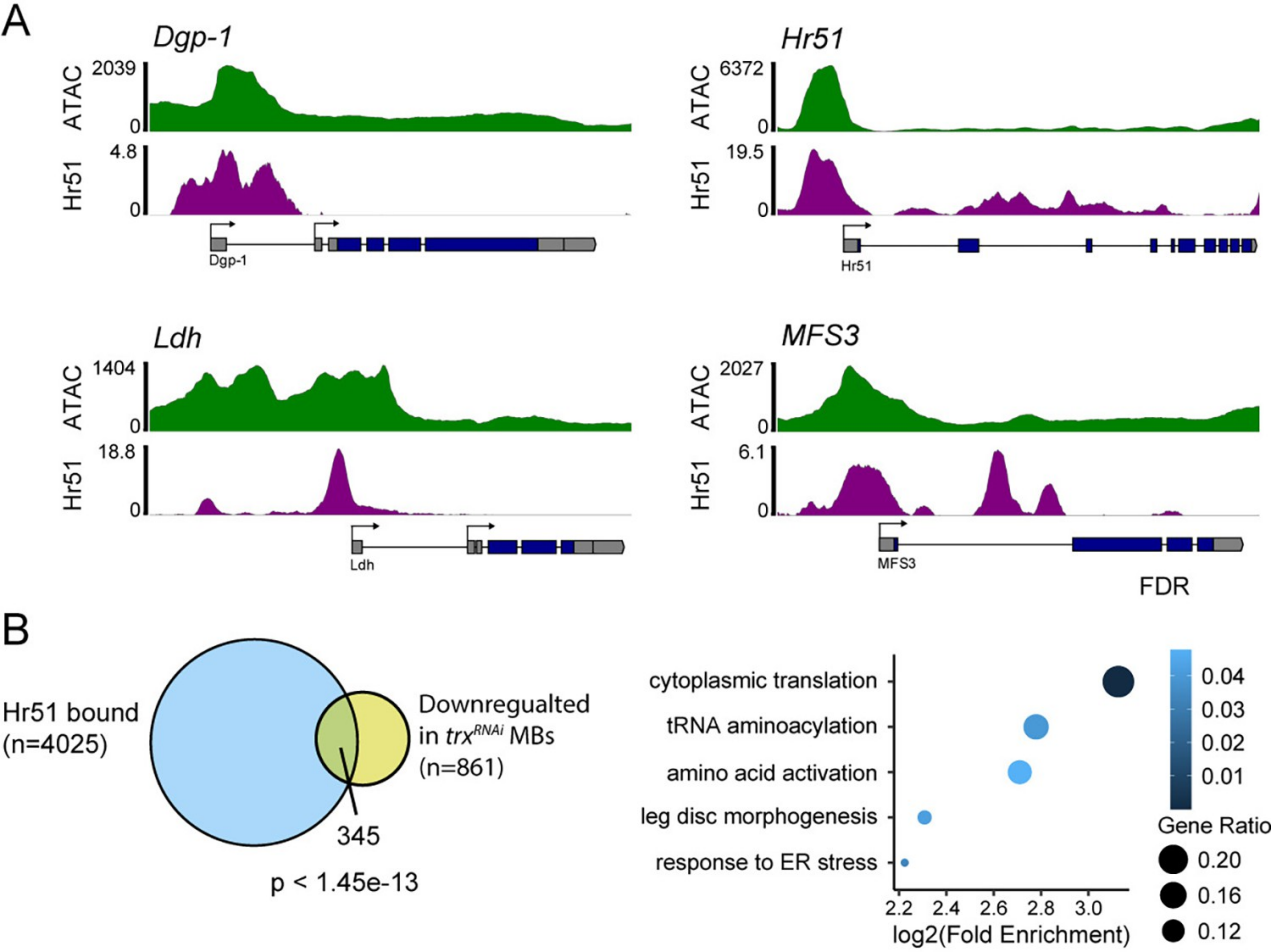
Hr51 target gene analysis. **(A)** Genome browser tracks showing chromatin accessibility (ATAC-seq–top panel), and Hr51 DNA binding sites (ChIP-seq–bottom panel) for 4 Trx target genes. Gene models are indicated below, with major alternative transcription start sites indicated by arrows. **(B)** Venn diagram showing the overlap between Hr51-bound genes and down-regulated genes following MB-specific *trx*^*RNAi*^. Overlap statistics assume a population of 13,986 coding genes in the *Drosophila* genome. Statistical significance was determined using a hypergeometric test. Adjacent bubble plot summarizes gene ontology enrichment analysis of the 345 overlapping genes. The 5 most enriched and representative terms are shown. Dot color indicates FDR, and size indicates the ratio of genes found in our gene list compared to the reference. Statistical significance was determined using Fisher’s exact test. MB, mushroom body.

Next, we sought to understand how MB-enriched Trx target genes support translation and LTM in the MB. Since Hr51 is a nuclear receptor transcription factor, we reasoned that it may activate important LTM genes downstream of, or in cooperation with, Trx. To investigate this, we used a published Hr51 ChIP-seq dataset (ENCSR555TTB) [73,74] to identify 4025 genes with Hr51 binding sites that overlap with MB-specific accessible regions, which we generated by INTACT ATAC-seq (**S3 Fig**). Among the Hr51 bound genes, we identified all 4 functionally validated MB-enriched Trx target genes, *Ldh*, *MFS3*, *Dgp-1*, and *Hr51* itself (**Fig 5A**). The Hr51 binding sites at these genes directly overlap with MB open chromatin and are adjacent to Trx binding sites and differential H3K4me3 peaks (**Figs 5A and 3D**). This suggests that Trx and Hr51 might work together to maintain expression of MB-enriched Trx target genes and that Hr51 is self-activating.

Interestingly, Hr51-bound genes are highly enriched for genes encoding structural ribosomal proteins, including 84 out of 177 genes annotated as structural constituents of the ribosome (GO:0003735). Hr51-bound genes also significantly overlap with genes down-regulated in *trx*^*RNAi*^ MBs (*p* < 1.45e-13) (**Fig 5B**). This includes 395 genes, which are enriched for GO terms related to translation, including cytoplasmic translation (GO:0002181), tRNA aminoacylation (GO:0043039), and amino acid activation (GO:0043038) (**Fig 5B**). In summary, this data suggests that Hr51 may act directly on some Trx target genes, including itself, and potentially also acts as a transcriptional activator of the translational machinery independent of Trx. Taken together with the observation that Hr51 is required for both STM and LTM in the adult MB, it appears that this orphan nuclear receptor may have a broader role regulating adult MB identity in a Trx-dependent and -independent manner.

**Fig 6:**
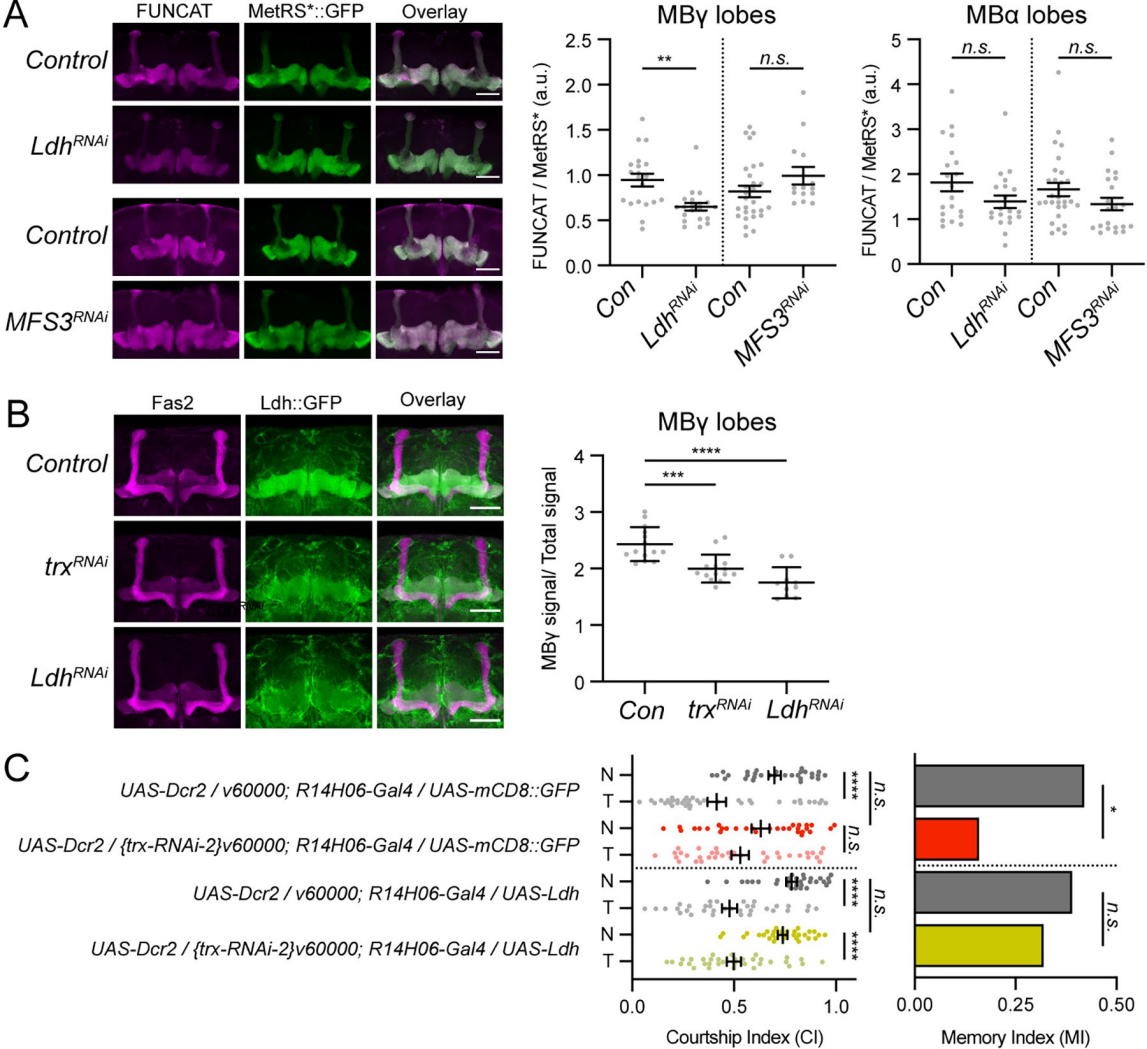
Ldh is required for LTM downstream of Trx. **(A)** Representative confocal slices showing FUNCAT signal (left panel), MetRS*::GFP (middle panel), and an overlay (right panel). Scale bars indicate 50 microns. Adjacent dot plots showing measurements of FUNCAT fluorescent intensity values normalized to MetRS*::GFP intensity in the MBγ (left) and MBα (right) lobes. MB-specific *Ldh*^*RNAi*^ (*UAS-Dcr2/+;R14H06-Gal4*,UAS-metRS*::*GFP/{Ldh-RNAi}v60000*, and *MFS3*^*RNAi*^ (*{MFS3-RNAi}attP40/+;R14H06-Gal4*,UAS-metRS*::*GFP/+*) strains were compared to genetic background controls (*UAS-Dcr2/+;R14H06-Gal4*,UAS-metRS*::*GFP/v60000* and *attP40/+;R14H06-Gal4*,UAS-metRS*::*GFP/+*, respectively). *P*-values were calculated using a Student’s *t* test. **(B)** Representative confocal stacks showing anti-Fas2 (left panel), Ldh::GFP (middle panel), and an overlay (right panel). Scale bars indicate 50 microns. Adjacent dot plots show normalized Ldh::GFP signal in MBγ lobes compared between control (*Ldh*::*GFP/v60000;R14H06-Gal4/UAS-Dcr2*), *trx*^*RNAi*^ *(Ldh*::*GFP/{trx-RNAi-2}v60000; R14H06-Gal4/UAS-Dcr2)*, and *Ldh*^*RNAi*^ (Ldh::GFP/UAS-Dcr2; R14H06-Gal4/{Ldh-RNAi-1}v60000) strains. *P*-values were calculated using a Student’s *t* test. **(C)** Courtship Indices (CIs—dot plots) for naive (N) or trained (T) flies and memory indices (MIs–bar graphs) for control flies (grays) and MB-specific *trx*^*RNAi*^ flies expressing *UAS-mCD8*::*GFP* (red), or *UAS-Ldh* (yellow). Full genotypes are indicated. *P*-values for comparison of N and T groups were calculated using the Mann–Whitney test. *P*-values comparing MIs between control and knockdown genotypes were calculated using a randomization test with 10,000 bootstrap replicates. *n*.*s*. not significant, *p < 0.05, **p < 0.01, ***p < 0.001, ****p < 0.0001. Raw data associated with this figure are available in **S1 Data**. LTM, long-term memory; MB, mushroom body; MI, memory index.

We next asked if the other MB-enriched Trx target genes, *Dgp-1*, *Ldh*, and *MFS3*, might be involved in supporting the translation capacity of MB neurons. Dgp-1 is the ortholog of Gtpbp1, which is required in mouse neurons to resolve ribosomal stalling during translation elongation [75]. In principle, Dgp-1 might influence translation downstream of Trx. In contrast, Ldh and MFS3 are not functionally linked to translation. Therefore, we performed FUNCAT on *Ldh*^*RNAi*^ and *MFS3*^*RNAi*^ MBs to test a possible role in translation. Interestingly, MB-specific *Ldh*^*RNAi*^ reduced translation in MBγ neurons, but not in MBα neurons (**Fig 6A and S1 Data**), fitting with the MBγ-specific expression of Ldh protein. *MFS3*^*RNAi*^ in the MB did not affect translation capacity in MBα or MBγ lobes (**Fig 6A and S1 Data**).

Considering that Trx and Ldh are both required for MBγ neuron translation capacity, LTM, and not STM, we tested if Ldh might be a limiting factor in the regulation of LTM downstream of Trx. MB-specific *trx*^*RNAi*^ results in decreased levels of Ldh::GFP protein in the MB, similar to that observed with *Ldh*^*RNAi*^ (**Fig 6B and S1 Data**). To replenish Ldh protein in *trx*^*RNAi*^ MBs, we co-expressed *UAS-Ldh*. Expression of Ldh did restore normal LTM in MB-specific *trx*^*RNAi*^ flies, while co-expression of *UAS-mCD8*::*GFP* did not (**Fig 6C and S1 Data**). These data show that Ldh expression in the MB is a limiting factor for MB translation capacity and LTM downstream of Trx.

### Trx and Ldh maintain a pool of lactate in MBγ lobes

**Fig 7:**
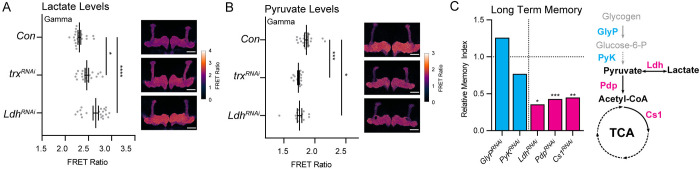
Trx and Ldh regulate the metabolic state of MBγ lobes. Dot plots showing FRET ratio in MBγ lobes for laconic (**A**) and pyronic (**B**) in flies with MB-specific *trx*^*RNAi*^, *Ldh*^*RNAi*^, and genetic background controls. FRET ratio was calculated as the YFP/CFP signal observed after correction for background fluorescence using a linear unmixing algorithm. The *P*-values were calculated using Student’s *t* test. The adjacent representative confocal slices show differences in FRET ratio corresponding to the control (upper panel), *trx*^*RNAi*^ (middle panel), and *Ldh*^*RNAi*^ (bottom panel). Scale bars indicate 50 microns. **(C)** Bar graphs showing the relative MIs of flies with MB-specific knockdown of genes involved in glucose metabolism (blue) and pyruvate’s entry into the TCA cycle (magenta). Horizontal dotted line indicates the relative levels of control MIs. *P*-values were calculated using a randomization test with 10,000 bootstrap replicates. Raw data and full genotypes of MB-specific knockdown strains and their genetic background controls are shown in **S5D Fig and S5 Table**. To the right, a schematic diagram highlights the metabolic functions of targeted enzymes involved in glucose metabolism (blue) and entry of pyruvate into the TCA (magenta). All raw data associated with this figure are available in **S1 Data**. MB, mushroom body; MI, memory index.

Ldh is required for the reversible conversion of lactate to pyruvate [76]. In mammals, the directionality of this conversion is thought to depend on the isozyme confirmation of LdhA and LdhB tetramers [77,78]. Unlike in mammals, *Drosophila* only has a single enzyme to perform both reactions, and how the directionality is determined is not well understood [76]. To understand how reduced Ldh protein might impact MB metabolism, we assessed levels of lactate and pyruvate in the MB lobes using Gal4-inducible laconic and pyronic FRET sensors, respectively [31,79]. The laconic and pyronic FRET sensors lose FRET activity upon lactate or pyruvate binding. Brains expressing laconic in the MB that were bathed in 40 mM lactate showed a decreased FRET intensity in the MB compared to brains bathed in PBS alone, demonstrating that our methods are able to detect changes in lactate concentration in the MB (**S5A Fig and S1 Data**). In *trx*^*RNAi*^ and *Ldh*^*RNAi*^ MBs, we observed that laconic FRET signals were higher in the MBγ lobes than in controls, indicating that MBγ lactate levels were reduced by depletion of Trx or Ldh (**Fig 7A and S1 Data**). Pyronic FRET signal was lower in *trx*^*RNAi*^ and *Ldh*^*RNAi*^ MBγ lobes, indicating a corresponding increase in the abundance of pyruvate (**Fig 7B and S1 Data**). No changes in lactate or pyruvate levels were found in the MBα lobes (**S5B Fig and S1 Data**), a part of the MB which does not have clear Ldh protein expression (**Fig 4C**). *MFS3*^*RNAi*^ did not impact lactate levels in the MB (**S5C Fig and S1 Data**). Taken together, these data show that Trx and Ldh are required in the MBγ lobe to maintain a pool of lactate.

### Energy metabolism genes are required for courtship LTM

It has been demonstrated that during *Drosophila* olfactory memory, up-regulated energy metabolism during memory consolidation is necessary and sufficient for LTM, but not required for STM [31]. LTM formation is known to consume large amounts of energy [80], likely due to the energy intensive cellular processes that are required for LTM, including transcription, translation, and maintenance of membrane potentials [81,82]. By blocking various critical steps in the metabolism of pyruvate through the TCA cycle, aversive olfactory memory is inhibited [31]. Interestingly, in this context, the pyruvate that is needed to feed the TCA cycle is not derived from glycolysis, but from alanine that is imported into the MBα neurons from glia [34]. Rationally, courtship conditioning likely also needs energy to support the taxing translation required for LTM. The stored lactate in MBγ neurons could provide an immediate source of pyruvate to feed the TCA cycle. Nevertheless, the role of metabolism in courtship LTM has not been explored.

To test the role of metabolic pathways in courtship LTM, we performed MB-specific knockdown of critical metabolic enzymes involved in glucose and pyruvate metabolism (**Fig 7C and S1 Data**). RNAi lines targeting these enzymes were validated in previous studies [31,33,83,84] and were shown in our hands to induce lethality when crossed to a ubiquitous *Act-Gal4* driver. GlyP is responsible for the breakdown of glycogen, a carbohydrate storage molecule, into glucose-6-P [85]. MB-specific *GlyP*^*RNAi*^ did not affect courtship LTM (**Figs 7C and S5D and S1 Data**), which was expected, since neurons are not typically thought to store glycogen [86]. Pyruvate kinase (PyK) is a critical enzyme that catalyzes the last step of glycolysis to convert phosphoenolpyruvate and ADP into pyruvate and ATP. MB-specific *PyK*^*RNAi*^ did not affect courtship LTM (**Figs 7C and S5D and S1 Data**), in agreement with other studies that suggest glycolysis is not essential for MB memory function [31,34]. For pyruvate to enter the TCA cycle and generate ATP, it must be converted into acetyl-CoA by pyruvate dehydrogenase (Pdh). Pdh is inhibited by Pdh kinase and activated by Pdh phosphatase (Pdp) [87]. MB-specific *Pdp*^*RNAi*^ reduced courtship LTM (**Figs 7C and S5D and S1 Data**), but not STM (**S5E Fig and S1 Data)**. Citrate synthase (Cs1) converts acetyl-CoA into citrate in the first and rate limiting step of the TCA cycle [88]. MB-specific *Cs1*^*RNAi*^ also reduced courtship LTM (**Figs 7C and S5D and S1 Data**), but not STM (**S5E Fig and S1 Data**). These data suggest that during courtship LTM, MB neurons do not use glycogen or glycolysis as an energy supply (gray lines, **Fig 7C**), but do rely on metabolism of lactate and pyruvate through the TCA cycle (black lines, **Fig 7C**). Since this analysis relies on a single RNAi line, it is possible that RNAi constructs that do not induce memory defects, for example, *GlyP*^*RNAi*^ or *PyK*^*RNAi*^, do not induce sufficient knockdown in the MB to manifest a phenotype. Therefore, we cannot conclusively rule out a role for glycolysis in courtship LTM. Despite this, these results do suggest that courtship LTM has a high energy requirement that is not needed for STM. This supports the idea that activation of *Ldh* by Trx facilitates LTM by enabling high energy capacity in MBγ neurons.

## Discussion

Trx is a classic epigenetic regulator that targets and activates hox genes to coordinate cell lineage specification during development [65,89,90]. However, the role of Trx post-development in adult tissues has not been studied. Here, we define a role for Trx in the adult MB of *Drosophila*. Loss of Trx in the MB causes deficits in LTM and translation capacity, but not STM (**Figs 1 and 2**). We show that Trx is required to maintain homeostatic expression of several novel MB-enriched transcripts that have expression levels similar to known MB identity genes (**Figs 3 and 4**). Based on their expression (**Fig 4A**), and their role in LTM (**Fig 4E**), we propose that these are novel MB identity genes that are critical for some, but not all, aspects of MB function. As an example, we show that the Trx target gene *Ldh* encodes a protein that is specifically expressed in the MBγ and not the MBα/β or MBα’/β’ neurons. Ldh supports the capacity of MBγ neurons for protein translation (**Fig 6A**) and is a limiting factor for LTM formation downstream of Trx (**Fig 6C**). Trx and Ldh also both contribute to a pool of lactate that is present in the MBγ neurons (**Fig 7A**). Based on the requirement for TCA cycle enzymes in courtship LTM (**Fig 7C**), we hypothesize that the MBγ lactate pool might feed the TCA cycle during LTM consolidation. This work identifies an unexpected role for Trx in homeostatic maintenance of metabolic state in MBγ neurons and supports the emerging idea that metabolic state is an important aspect of cell identity that is genetically encoded in neurons that form LTM.

### Metabolic state as a Trx-dependent aspect of postmitotic neuron cell identity

Neuron identity is defined by a combination of different characteristics, including neurotransmitter type (e.g., cholinergic, GABAergic), morphology, location, and the presence of different types of cell surface receptors [1,2]. Many neuron types also have specialized functions that require additional specific cellular machinery [2]. Neuron cell identity characteristics are usually established through the expression of a battery of effector genes, often called a subroutine. For example, the cholinergic gene battery includes expression of several enzymes and other specialized proteins to produce acetylcholine (e.g., choline acetyltransferase) and package it into vesicles (e.g., vesicular acetylcholine transporter) [2,3]. Neuron cell identity features are maintained by terminal selector TFs that continuously activate the expression of the required effector genes [6,91]. In *Drosophila*, some TFs regulate broad aspects of cell identity [92,93], whereas others only activate specific subroutines [15,17,18]. One example of this is illustrated in *Drosophila* secretory neurons of the larval brain, where a specific TF, Dimm, activates expression of a general neurosecretory subroutine which includes proteins involved in vesicle biology [15,17,18]. The full repertoires of cell identity subroutines and the TFs that control them are not known.

Our data suggest that the epigenetic regulator Trx may facilitate a metabolic subroutine in MB neurons that supports the capacity of these neurons to form LTM. We show that Trx maintains high MB expression levels of several transcripts involved in metabolism and translation (**S4 Table**). Beyond *Hr51* and *Ldh*, additional MB-enriched Trx target genes include *Dgp-1*, potentially involved in translation [75], metabolite transporters *MFS3* and *JhI-21* [94,95], and *Xrp1*, a protein that is involved in DNA damage response and activated in translation deficient cells [96]. Overall, this supports the idea that Trx facilitates an MB metabolic subroutine required for LTM formation. Interestingly, it has been shown that during LTM consolidation in an aversive olfactory memory assay, the MB, and not the surrounding brain tissues, undergoes a high level of pyruvate flux directed into the TCA pathway for mitochondrial ATP generation [31]. Forced activation of this MB-specific energy flux facilitated the formation of LTM under training conditions that would normally not induce LTM [31]. This further supports the idea that metabolic capacity is a critical feature of MB neuron identity that facilitates the capacity to form LTM. Our findings demonstrate that some aspects of MB metabolic state are mediated by epigenetic maintenance of MB-enriched transcripts, through Trx.

### Alternate energy strategies genetically encoded in MB subtypes

In *Drosophila* aversive olfactory memory, MBα/β neurons import alanine, which is converted to pyruvate to feed the TCA cycle during LTM consolidation [34]. Here, we show that disrupting the entrance of pyruvate into the TCA cycle also impairs courtship LTM (**Fig 6D**), suggesting that the requirement for mitochondrial ATP generation may be conserved between olfactory and courtship LTM. However, there appears to be key genetically encoded differences between the MBα/β neurons that underly olfactory memory, and the MBγ neurons that underly courtship LTM. Specifically, we show here that Ldh protein is expressed in the MBγ neurons but not the MBα/β and α’/β’ neurons. In steady state conditions, Trx and Ldh help to promote or maintain a high lactate to pyruvate ratio MBγ lobe (**Fig 7A and 7B**). Accordingly, *Drosophila* olfactory memory does not appear to require Ldh [34], while courtship LTM does (**Fig 4E**). Taken together, it appears that different MB neurons have different metabolic identities that are genetically encoded.

### Lactate metabolism in long-term memory

Why do MBγ neurons accumulate lactate and what is it used for? Several possibilities exist, the most probable may be that lactate is stored as a critical energy source to feed the TCA cycle during LTM formation. LTM consolidation in MBα/β neurons requires imported alanine, which is converted to pyruvate as a source of energy, feeding the TCA cycle [34]. In MBγ neurons, the external energy supply is not yet known. We show that MBγ neurons use Ldh to store lactate. Lactate is typically seen as a waste product resulting from incomplete oxidation of glucose, but recent work suggests that it may have a more prominent role as an energy storage molecule (reviewed in [97]). Lactate can be converted to pyruvate by Ldh in a chemical reaction that requires the conversion of NAD+ to NADH, a critical provider of electrons for mitochondrial ATP production. Two molecules of lactate converted to pyruvate generate equivalent energy as when glucose is converted to pyruvate through glycolysis. In contrast, pyruvate and alanine, other MB energy sources, represent more oxidized carbon forms with less energy potential. Carbon tracing experiments in rodents suggest that lactate, rather than glucose, is the primary energy transport molecule that feeds the TCA in many mammalian tissues [98,99]. We hypothesize that MBγ neurons also take advantage of lactate as a superior energy storage molecule that is used during courtship LTM formation. The use of lactate as a critical energy metabolite for *Drosophila* LTM has been proposed, but not clearly demonstrated [100]. In contrast, the use of lactate as a critical neuronal energy source is well studied in mammalian models in a process known as the astrocyte-neuron lactate shuttle (ANLS) [101,102]. In rodents, astrocytes breakdown stores of glycogen to produce lactate, which is then shuttled to neurons, where it is converted into pyruvate to supply the TCA cycle. Glial export of lactate and the uptake of lactate in neurons are necessary for rat avoidance LTM [103], and also support de novo translation in excitatory and inhibitory neurons of the rat dorsal hippocampus [82]. Translation is an energy intensive process which consumes nearly 35% of a cell’s available ATP [104]. It was therefore suggested that the high ATP cost of translation during LTM formation is sustained by increased TCA cycle activity [82]. Our finding that Ldh supports LTM (**Fig 4E**), translation (**Fig 6A**), and lactate levels (**Fig 7A**), suggests a similar mechanism may occur in MBγ neurons.

While it is probable that the lactate pool maintained by Trx and Ldh in MBγ neurons is used to supply the TCA cycle to produce ATP for translation in the context of LTM, it is not currently possible to directly test this by measuring lactate flux during courtship memory consolidation. Therefore, we must consider other possible uses and consequences of lactate storage in neurons. For example, lactate can affect cell signaling by acting as an agonist for the G-protein coupled HCAR1 receptor and can modulate neuronal activity in mammals [105,106]. It is therefore possible that lactate may contribute to LTM through a role in signaling within the MB. Lactate may also influence gene expression through effects on chromatin. Recently, it was shown that neural excitation and social stress correspond to changes in Histone H1 lactylation in mouse neurons, which correlated with increased expression of *c-Fos*, an early marker for neuronal activity [107]. Histone lactylation is a recently discovered chromatin modification that is not well understood, and no study has yet identified direct gene targets of histone lactylation in neurons. In addition, lactate might also affect gene expression indirectly through its link to acetyl-CoA and NADPH. A high lactate to pyruvate ratio would result in low levels of acetyl-CoA, which is required for gene activating histone acetylation. The generation of lactate also requires NADH to be converted to NAD+, which is a cofactor for NAD+-dependent class histone deacetylases [108]. This combination would in theory lead to low histone acetylation, which is associated with low transcription. Considering these possibilities, we cannot rule out a multifunctional role for lactate in MBγ neurons at this time.

### Hr51 is a candidate terminal selector in the adult MB

The role of Trx in Ldh expression is likely facilitative, as *trx* knockdown does not completely abolish Ldh expression (**Fig 3C**). Single-cell RNA-sequencing from whole *Drosophila* shows that Trx is ubiquitously expressed in all tissues [109], and therefore cannot be instructive for Ldh activation. A question that arises from this is how a ubiquitously expressed factor like Trx can have cell-type specific functions. Hr51 is one MB-enriched gene that may be an instructive factor for adult MB subtype identity. During pupal developmental MBγ neuron axons are entirely pruned back and then re-extend to form the adult MBγ lobe [71,72]. Without Hr51, MBγ neuron remodeling can stall, causing the final adult neuron to have an incorrect morphology. Key neuronal identity markers are also lost in adult MB neurons in the absence of Hr51, including trio (specific for MBα’/β’ and MBγ), and Fas2 (specific for MBα/β and MBγ neurons) [72]. In addition, the data presented here shows that Hr51 has other hallmarks of terminal selectors [1]. With Hr51 ChIP-seq data, it was shown that Hr51 has the capacity for autoregulation by binding to its own promoter, and binding to the promoter of additional MB enriched transcripts identified in this study (**Fig 5A**). Hr51 protein is also highly and very specifically expressed in the nuclei of MB neurons, and in few other cells of the central brain (**Fig 4D**) [70]. In this study, courtship conditioning revealed that Hr51 is required in adult MB neurons to facilitate STM and LTM (**Fig 4E**) [72], suggesting that it likely has a broader role than Trx in defining MB identity.

## Conclusions

Overall, we show that Trx has a specific role in regulation of LTM in the adult *Drosophila* MB that is not redundant with the other *Drosophila* H3K4 methyltransferases, Set1 and Trr. Trx targets several novel MB enriched genes involved in translation and metabolism. As a result, Trx-deficient MB neurons show reduced translation capacity and altered metabolic state. Trx regulates MB metabolic state through a key target gene, Ldh, which promotes lactate storage under steady state conditions. Based on the importance of lactate as an energy storage molecule and the known role of glycolysis-independent TCA cycle activity in LTM, we hypothesize that lactate serves as an energy storage pool in MBγ neurons. Trx, therefore, is required to maintain the metabolic identity of adult MBγ neurons through histone methylation of novel MB-identity genes.

## Methods

### *Drosophila* stocks and genetics

Unless otherwise stated flies were reared on a standard media (cornmeal-sucrose-yeast-agar) at 25°C and 70% humidity with a 12 h:12 h light/dark cycle. *Drosophila* stocks were acquired from the Bloomington *Drosophila* Stock Center (BDSC), the Vienna *Drosophila* Resource Center (VDRC), the Kyoto Stock Center (KSC), FlyORF, or donated from other labs. *R14H06-Gal4* flies express Gal4 under the control of a MB-specific enhancer for the adenyl cyclase gene *rutabaga* (BDSC #48677) [42]. UAS-RNAi and ORF stock lines used in this study include: *trx*^*RNAi*^ (1 = BDSC #31092 & 2 = VDRC #37715), *Mnn1*^*RNAi*^ (1 = VDRC #17701 & 2 = VDRC #110376), *trr*^*RNAi*^ (1 = BDSC #29563 & 2 = VDRC #110276, 3 = BDSC 36916), *Set1*^*RNAi*^ (1 = BDSC #33704 & 2 = BDSC #38368) *Ldh*^*RNAi*^ (1 = VDRC #31192 & 2 = BDSC #33640), *MFS3*^*RNAi*^ (VDRC #330237), *Hr51*^*RNAi*^ (BDSC #39032), *Dgp-1*^*RNAi*^ (VDRC #27490) *GlyP*^*RNAi*^ (VDRC #109596), *Cs1*^*RNAi*^ (VDRC #26301), *Pdp*^*RNAi*^ (VDRC #31661), *PyK*^*RNAi*^ (VDRC #49533), and *UAS-Ldh* (FlyORF, F002924). *UAS-Unc84*::*GFP* flies were a gift from Lee Henry [53]. Fluorescent labeling of MB cell membranes was done with *UAS-mCD8*::*GFP* (BDSC #5130 & #5137). UAS-MetRS*::*GFP* flies were a gift from Elaheh Soleimanpour [55]. Flies with *UAS-Laconic* and *UAS-Pyronic* FRET sensors were a gift from Pierre-Yves Plaçais [31,110]. Fluorescently tagged proteins used in this study include: Ldh::GFP, gifted by Erica Geisbrecht [68], MFS3::YFP (KSC #118654), Hr51::GFP (BDSC #38650), and Dgp-1::GFP (this paper). The Dgp-1 tagged protein was generated using the CH322-96L23 bacterial artificial chromosome (BAC) containing the *Dgp-1* gene (chr2R from 18165512 to 18183054) from the P[acman] BAC library [111]. This BAC was incorporated into SW102 *E*. *coli* cells. A multi-tag including SGFP, 3xFLAG, and V5 was amplified from FlyFos022810 DNA. Recombineering was done to insert the multi-tag directly before the stop codon at the end of the *Dgp-1* gene within the CH322-96L23 BAC, as described [112]. This construct was verified through Sanger Sequencing. The modified BACs were then isolated and sent to Genome Prolab for the creation of a transgenic fly with the tagged *Dgp-1*::*2XTY1*::*SGFP*::*V5*::*preTEV*::*BLRP*::*3xFLAGdFRT* transgene.

For RNAi knockdown experiments, we used a heterozygous isogenic breeding strategy to control for genetic background. In all crosses, a Gal4 line was used containing *R14H06-Gal4*, either alone, or combined with different accessory transgenes; including *UAS-Dicer-2*, *tubP-Gal80*^*ts*^, *UAS-mCD8*::*GFP*, UAS-MetRS*::*GFP*, *UAS-laconic*, *UAS-pyronic*, and *UAS-Unc84*::*GFP*. To produce RNAi and control genotypes, Gal4 lines were crossed to (1) a UAS-RNAi line; and (2) a line that was isogenic to the UAS-RNAi line. For several crosses, UAS-RNAi transgenes were combined with additional transgenes, and in these cases an isogeneic control was created using chromosomes from the appropriate genetic background strain, either VDRC #60000 (*v60000*), VDRC #60100 (*attP30B*,*attP40D*), BDSC #36303 (*attP2*), BDSC #35785 (*mCherry{attP2}*), or BDSC #36304 (*attP40*). All crosses, including isogenic parental and test genotypes are shown in **S5 Table**.

Gal80^ts^ was used to induce temporal control of RNAi knockdown. Gal80^ts^ inhibits Gal4 at 18°C but is inactive at 29°C. To induce larval/pupal knockdown, flies were incubated at 29°C from egg laying to induce expression of *UAS-RNAi* transgenes and moved to 18°C immediately following eclosion to inhibit the *UAS-RNAi* expression. The opposite temperature shift protocol was used to induce adult-specific knockdown (**S1B and S4F Figs**). In all experiments using Gal80^ts^, isogenic genetic background controls with *Gal4/Gal80* and no *UAS-RNAi* transgene, as well as flies with a heterozygous *UAS-RNAi* transgene and no *Gal4/Gal80*^*ts*^ were subjected to the same temperature shift protocols. In all cases, these controls were observed to have normal memory or neuronal morphology indicating that observed memory phenotypes were not induced by temperature changes.

### Courtship conditioning assay

STM and LTM were assessed using courtship conditioning, as previously described [27,43,44]. Briefly, naïve male flies were trained by pairing with an unreceptive premated female for 1 h (STM) or 7 h (LTM). After a rest period of 1 h (STM) or 20 h (LTM), males were paired with a new premated female and a courtship index (CI) was calculated, which is the proportion of time spent courting over 10 min. CIs of trained flies were compared to CIs of naïve flies that were not exposed to a female using a Mann–Whitney test. The memory index (MI) represents the percentage reduction in courtship behavior in trained flies compared to naive and is used to compare memory between different genotypes. MI was calculated using the formula: MI = ($\bar{x}$ CI_naive_ - $\bar{x}$ CI_trained_) / $\bar{x}$ CI_naive_. *P*-values comparing MIs were calculated using a randomization test with 10,000 bootstrap replicates, as described previously [27,44].

### Immunohistochemistry

Brains were dissected in PBS and fixed with ice cold 4% paraformaldehyde for 30 to 45 min. For immunohistochemistry, fixed brains were blocked in 5% NGS then incubated overnight with the primary antibodies anti-GFP (1:100, Invitrogen: G10362), anti-Fas2 [1:25, Developmental Studies Hybridoma Bank (DSHB): #1D4], or anti-dac (1:100, DSHB: mAbdac2-3), and secondary antibodies AlexaFluor 488 or 594 (1:300, Invitrogen: A1108 & A1105). Brains were then mounted in SlowFade Antifade (Invitrogen: S36972) or VectaShield (VectorLabs: H-1900) before imagining. Images were acquired using a Zeiss LSM 510, 710 or 880 confocal microscopes. Confocal stacks were processed and quantified using ImageJ software [113].

### Sleep, circadian behavior, and activity analysis

The activity assay and sleep analyses were conducted as previously described [27]. Briefly, flies (0 to 3 days old) entrained in light/dark cycles (LD; 12 h:12 h) for 3 days are loaded into cuvettes with solid fly food at one end. Flies are monitored for locomotion using *Drosophila* Activity Monitor 5M (DAM5M) (TriKinetics Inc, Massachusetts, United States of America) at 25°C LD for 2 full days and subsequently in constant darkness (DD) for 7 days, totalling 9 full days of activity recording at 1-min resolution. In calculating sleep behavior in LD, 5 min of continuous inaction was considered a sleep bout [114,115] and quantified using locally written python code. Circadian behavior in DD was analyzed using the ActogramJ plug-in in ImageJ [116] using Lomb-Scargle.

### Gene Ontology enrichment analysis

Gene Ontology (GO) enrichment analysis was done using PANTHER (version 17.0) [117–120]. For the different sets of gene lists, biological processes were considered with an adjusted *p*-value cut-off of 0.05, using Fisher’s exact test with FDR multiple test correction. For genes that were induced after training, we displayed the most enriched term from the hierarchical branches of the GO term network. For all other GO term analyses, only the most enriched terms with between 25 and 250 genes in the reference genome were included. Semantically redundant terms were excluded, including only the most significant term.

### Fluorescent non-canonical amino acid tagging (FUNCAT)

Flies were prepared for ANL labeling as previously described [55]. In brief, adult flies were reared on 10 mM ANL supplemented sucrose-agar food for 4 days before dissections. Fixed brains were treated with a FUNCAT-reagent overnight, and then stained with 1:500 anti-TAMRA (Invitrogen: MA1-041) and 1:100 anti-GFP (Invitrogen: G10362). Secondary staining and mounting was done as described above. Brains were imaged on a Zeiss LSM 880 microscope and measurements were taken as a mean intensity from the middle slice of the MBγ lobes.

### Feeding assay and analysis

The Activity Recording Capillary Feeder (CaFe) assay was previously described [121]. Briefly, the feeding arena is 3D-printed in acrylic, and 1% (wt/vol) agar was placed at the bottom of each well to allow ad libitum hydration. Liquid food delivered in borosilicate capillaries (VWR: 53432–706) was composed of 2.5% (w/v) Bacto yeast extract (BD: 212750) and 2.5% (w/v) sucrose (Sigma-Aldrich: 57-50-1) and stored at −20°C. Tracking dye (dodecane: mineral oil, 3:1) (Sigma-Aldrich: 297879 and 330779) containing copper reagent (Sigma-Aldrich: 415286) was placed at the top of the liquid food as a tracking marker. Data was analyzed as previously described [121]. Total food consumed over 24 h was summed for each fly across all bouts and compared between genotypes using Student’s *T* test.

### Isolation of nuclei tagged in a specific cell type (INTACT)

Mushroom body nuclei were isolated with INTACT, as previously described, from a pool of 50 to 70 male flies per replicate [40]. Briefly, fly heads expressing *UAS-Unc84*::*GFP* with the MB-specific driver, *R14H06-Gal4* [42], were ground with a pestle in a 1.5 ml tube, and homogenized with buffer containing 0.3% NP40 in a Dounce homogenizer. Nuclear extract was passed through a 40 μm cell strainer. Unc84::GFP labelled nuclei were then immunoprecipitated with anti-GFP antibody (Invitrogen: G10362) bound to magnetic beads (Invitrogen: 10004D), according to the manufacturer’s instructions.

### RNA-sequencing and data analysis

RNA-sequencing was performed on INTACT isolated MB nuclei from flies with MB-specific *trx*^*RNAi*^ (*N* = 7), *trr*^*RNAi*^ (*N* = 5), *or Set1*^*RNAi*^ (*N* = 4), and 2 genetic background control lines (*N* = 8 and *N* = 5) (GSE239493). RNA-seq libraries from *trx*^*RNAi*^ MBs (*UAS-unc84*::*GFP/{trx-RNAi-2}v60000;R14H06-Gal4/UAS-Dcr2*) and their genetically matched controls (*UAS-unc84*::*GFP/v60000;R14H06-Gal4/UAS-Dcr2*) were generated as previously described [40]. In brief, RNA from INTACT-isolated MB nuclei was isolated with the PicoPure RNA isolation Kit (Invitrogen: KIT0204), and libraries were prepared with the Nugen Ovation *Drosophila* RNA-Seq System 1–16 Kit (Nugen: NU035032). Sequencing was performed with the NextSeq500 at the London Regional Genomics center with Illumina high output v2 75 cycle kit to a read length of 75 single end base pair reads. RNA-seq libraries for MB *trr*^*RNAi*^ and *Set1*^*RNAi*^ flies (*UAS-unc84*::*GFP;{trr-RNAi-3}attP2/R14H06-*Gal4 and *UAS-unc84*::*GFP;{Set1-RNAi-1}attP2/R14H06-Gal4*) and their genetically matched control (*UAS-unc84*::*GFP;{mCherry-RNAi}attP2/R14H06-Gal4*), were prepared using the Tecan Universal Plus Total RNA-Seq library preparation kit (0361-A01) according to manufacturer’s instructions and sequencing was performed with the Illumina NovaSeq 6000 at Genome Quebec with the S4 v1.5 200 cycle kit (100 bp paired end reads).

Raw sequence reads were trimmed to a minimum base quality of 30 using Prinseq (version 0.20.4) [122] or Trimmomatic (v0.39) [123]. Trimmed reads were aligned using STAR (version 2.5.3a and version 2.7.5a) to the *Drosophila* genome (BDGP release 6) [124–126]. Reads that aligned to multiple loci, or to one locus with >4 mismatches, and genes that mapped to *Drosophila* rRNA were removed. Following alignment, samples had on average 25 million useable reads remaining, with no fewer than 15 million reads in any sample. Gene counts were obtained by HTSeq-count (version 0.7.1) using the default union setting or using FeatureCounts from the Rsubread R package (version 2.4.2) [127]. Y-chromosome and mitochondrial genes, and genes with no reads were removed before differential expression analysis using DESeq2 (version 1.30.1). Differentially expressed genes were identified using DESeq2 to employ the Wald test with correction for multiple testing using the Benjamini and Hochberg method. Normalized counts used for comparing gene expression between conditions were generated using the median of ratios of DESeq2. Analysis of overlap between genes down-regulated in *trx*, *Set1*, and *trr* knockdown MBs was performed using the R Bioconductor package GeneOverlap [128].

### ChIP-sequencing and data analysis

H3K4me1 and H3K4me3 ChIP sequencing was performed on INTACT-isolated MB nuclei from flies with MB-specific knockdown of Trx, and genetic background controls. ChIP was performed using the True ChIP-seq Kit (Diagenode: C01010132) following manufacturers guidelines with the exception that bead-bound nuclei were fixed with 36.5% formaldehyde for 1 min. Fixed nuclei were then lysed for 10 min with lysis buffer and DNA was sheared with a Covaris M220 sonicator for 10 min. Immunoprecipitations were done with anti-H3K4me1 and H3K4me3 antibodies (Diagenode: C15410194 and C15410003). DNA was purified using MicroChIP DiaPure columns (Diagenode: C03040001), and libraries were generated with the MicroPlex Library Preparation Kit (Diagenode: C05010001). Completed libraries were sequenced on an Illumina NovaSeq 6000 S4 at Genome Quebec (100 bp paired end).

Raw sequence reads were trimmed using Trimmomatic (version 0.39) [123] and trimmed reads were aligned with Bowtie (version 2.4.1) using the very-sensitive setting to the *D*. *melanogaster* genome (BDGP release 6) [124,125,129]. Reads with >4 mismatches as well as reads aligning to mitochondrial chromosomes and scaffolds were removed. Following alignment, H3K4me3 samples had on average 60 million useable reads remaining, with no fewer than 45 million reads in any sample. H3K4me1 samples had on average 57 million useable reads remaining, with no sample having fewer than 15 million useable reads remaining. ChIP peaks were identified with the MACS2 (version 2.2.7.1) [130] callpeak function with a cut-off of *q* < 0.01. Peaks were filtered against the ENCODE blacklist to remove peaks occurring in regions with high occurrences of artifacts [131], and then used to generate a consensus peak set. Differential peak analysis was performed with DiffBind (version 3.0.15) [128]. Peaks were annotated with ChIPSeeker (version 1.26.2) [132]. ChIP data for Trx (GSE24521) was obtained from the GEO database [61]. ChIP data for Hr51 (ENCSR555TTB) was downloaded from the ENCODE portal [73]. ChIP and ATAC tracks were generated with bamCoverage from the deepTools program (version 3.5.1) [133], then averaged between replicates with WiggleTools (version 1.2) [134]. Averaged tracks were then visualized with pyGenomeTracks (version 3.6) [135].

### ATAC-sequencing and data analysis

ATAC-seq was performed as previously described [136] on INTACT-isolated MB nuclei from 2 independent biological replicates. Bead-bound nuclei were suspended in 50 μl of transposase reaction mix (Tn5 Transposase, Illumina) and incubated for 30 min at 37°C. DNA was then purified and eluted using a Qiagen MinElute Kit. Purified DNA was mixed with custom Nextera primers and High-Fidelity PCR Mastermix (NEB), amplified as per manufacturer’s instructions, and then purified using a Qiagen PCR purification kit. Sequencing was performed with the Illumina NovaSeq 6000 at Genome Quebec with the S4 v1.5 200 cycle kit; read length was 100 bp for paired-end reads.

ATAC-seq reads were trimmed using Trimmomatic (version 0.39) [123]. Trimmed reads were aligned to the *D*. *melanogaster* reference genome (BDGP release 6) using Bowtie2 (version 2.4.1) with the settings -X 2000 and–very-sensitive [125,129]. Reads were then shifted, +4 bp for the forward strand and −5 bp for the negative strand, to account for the 9-bp duplication created by DNA repair nick of the Tn5 transposase [137]. Reads aligning to multiple loci, the mitochondrial genome, and scaffolds were excluded. Duplicate reads resulting from PCR amplification were identified and removed leaving 133,163,624 and 57,951,806 high-quality reads for downstream analysis. Peaks were then identified in each replicate with MACS2 (version 2.1.2) using the settings -q 0.01 –min-length 50 and–max-gap 100 [130]. This identified 14,655 consensus peaks between the 2 replicates, which are found in or near 8,588 genes. Both libraries had FRiP (fraction of reads in peaks) scores greater than 0.3, as calculated by DiffBind. The 2 replicates also showed high consistency in fragment lengths and number of reads per peak **(S3 Fig)**, suggesting that the data was reproducible. For visualization of ATAC data, bam files were normalized using the bamCoverage function from deepTools with scale factors determined by the dba.normalize function from Diffbind.

### Metabolite analysis using FRET sensors

Adult male brains expressing *UAS-laconic* or *UAS-pyronic* in MB neurons with *R14H06-Gal4* [42] were dissected in PBS and fixed with ice cold 4% paraformaldehyde for 30 to 45 min. Mounted brains were imaged with a Zeiss LSM 880 to capture 3 relevant channels simultaneously after excitation at 458 nm. Emission windows of the channels are: donor (CFP) channel, 480–500 ± 5 nm; acceptor (YFP) channel, 525–545 ± 5 nm; and autofluorescence (AF) channel, 595–615 ± 5 nm. FRET quantification was done using a previously described linear unmixing algorithm [79] to correct for background and autofluorescence. We modified the published script using FIJI (version 2.3) [138] to perform linear unmixing on our MB images. Briefly, the linear unmixing algorithm corrects the CFP and YFP channels by adjusting them to the background signal in the AF channel. Correction involves dividing the CFP and YFP channels by the AF channel, then subtracting these ratios from the original CFP and YFP channels to create corrected CFP and YFP channels. Next, the corrected CFP channel is divided by the corrected YFP channel to create a ratiometric FRET value for every pixel in the image [79]. The mean FRET ratios of regions of interest were determined by measuring the corrected ratiometric values from a single 1 μm slice from the middle of the MBγ or MBα lobes, which encompasses the widest part of the lobes. Regions of interest were manually selected to include the entire visible area of the MBγ or MBα lobes.

## Supporting information

S1 FigRelated to Fig 1.**(A, B)** Courtship indices (CIs–dot plots) and memory indices (MIs—bar graphs) underlying relative MIs shown in **Fig 1A** and **1B**. The mean and SEM are indicated in dot plots. Full genotypes are indicated. Genetic background controls are represented using grays, MB-specific RNAi knockdown genotypes are indicated using colors. *P*-values comparing naive (N) and trained (T) flies were generated using a Mann–Whitney test. MIs are calculated from CIs using the formula: MI = ($\bar{x}$ CI_naive_ - $\bar{x}$ CI_trained_) / $\bar{x}$ CI_naive_. Statistical significance between MIs was determined using a randomization test with 10,000 bootstrap replicates. (**A**) Courtship STM (left panel) and LTM (right panel) was assessed upon knockdown of *Drosophila COMPASS* subunits, *Set1*, *trr*, *trx*, and *Mnn1*. Previously published data for Set1 [27] is shown for comparison. **(B)** Gal80^ts^ was used to restrict MB specific *trx*^*RNAi*^ and *Mnn1*^*RNAi*^ to the larvae/pupae stage or the adult stage. For larvae/pupae knockdown, MB RNAi flies and genetic controls were raised at 29°C and transferred to 18°C at eclosion to prevent RNAi expression in adults. For adult-specific knockdown, flies were raised at 18°C and shifted to 29°C at eclosion. **(C)** Ribbon plot showing average sleep per minute per fly of MB specific *trx*^*RNAi*^ flies (red: *UAS-Dcr2/{trx-RNAi-2}v60000; R14H06-Gal4/+*) compared to controls (blue: *UAS-Dcr2/v60000;R14H06-Gal4/+*), averaged over 48 h. Flies are considered asleep if they exhibit no activity over at least 5 min. Average sleep is calculated as the average of sleeping flies (value: 1) or awake flies (value: 0). Line thickness is mean sleep, +/- SEM. White and gray backgrounds indicate objective day and night, respectively. Vertical black lines indicate at least 2 contiguous blocks of statistically significant differences in sleep behavior, measured using Student’s *t* test. Raw data associated with this figure are available in **S1 Data**.(TIF)

S2 Fig Related to Fig 2. (**A**) Dot plots showing normalized expression values for: (1) training 0-induced genes identified in both control and *trx*^*RNAi*^ MBs (left panel), (2) training-induced genes identified only in control MBs (middle), and (3) training-induced genes identified only in *trx*^*RNAi*^ MBs (right). Control genotype: *UAS-unc84*::*GFP/v60000;R14H06-Gal4/UAS-Dcr2*. *trx*^*RNAi*^ genotype: *UAS-unc84*::*GFP/{trx-RNAi-2}v60000;R14H06-Gal4/UAS-Dcr2*. *P*-values were calculated using pairwise Wilcoxon tests. ****p < 0.*0001*. **(B)** Dot plot showing food consumed by *trx*^*RNAi*^ flies (red: *UAS-Dcr2/{trx-RNAi-2}v60000;R14H06-Gal4/+*) compared to controls (blue: *UAS-Dcr2/v60000;R14H06-Gal4/+*) over a 24-h period. Statistical significance was determined using Student’s *t* test. *n*.*s*.–not significant. Raw data associated with this figure are available in **S1 Data**.
(TIF)

S3 Fig Related to Fig 3. **(A)** Fragment size distribution of ATAC-seq libraries generated from INTACT-isolated MB nuclei. Distribution for 2 biological replicates is shown. Peak signal of nucleosome free regions (80–120 bp) and mononucleosomes (~180 bp) is marked. **(B)** Scatter plot of normalized counts for consensus peaks from MB ATAC-seq samples. Each dot represents a consensus peak, with positions indicating normalized counts in replicate 1 and replicate 2. Line of best fit is shown, with corresponding linear equation, and coefficient of determination. **(C)** Feature distribution of annotated ATAC-seq peaks, which are predominantly located near transcriptional start sites (TSS) of genes.
(TIF)

S4 FigRelated to Fig 4.**(A–D)** Confocal z-stack projections showing localization of **(A)** MFS3::YFP, **(B)** Dgp-1::GFP, **(C)** Ldh::GFP, and **(D)** Hr51::GFP (middle panels) in the **(A–C)** MB lobes (anti-Fas2 –left panel) or **(D)** MB nuclei (anti-dac–left panel). In **(A)** and **(B)** regions of interest are defined by a white box and shown immediately below. For **(C)** and **(D)** regions of interesting are defined by a white box and shown in **Fig 4C** and **4D**. Scale bars indicate 50 microns. **(E)** Courtship indices (CIs–dot plots) and memory indices (MIs-bar graphs) underlying relative MIs from **Fig 4E**. The mean and SEM are indicated in dot plots. Full genotypes are shown. Genetic background controls are represented using grays, MB-specific RNAi knockdown genotypes are indicated using colors. *P*-values comparing naive (N) and trained (T) flies were generated using a Mann–Whitney test. MIs were calculated from CIs using the formula: MI = ($\bar{x}$ CI_naive_ - $\bar{x}$ CI_trained_) / $\bar{x}$ CI_naive_). Statistical significance between MIs was determined using a randomization test with 10,000 bootstrap replicates. Raw data associated with this figure are available in **S1 Data. (F)** Confocal z-stack projections showing the impact of *Hr51*^*RNAi*^ on MB morphology. Unrestricted knockdown (i.e., without Gal80^ts^) of *Hr51* in the MB with *R14H06-Gal4* at 25°C (UAS-MetRS*::*GFP/+;{Hr51-RNAi}attP2/R14H06-Gal4*) led to loss of MBγ lobes that was not observed in controls (UAS-MetRS*::*GFP/+;{mcherry-RNAi}attP2/R14H06-Gal4*). When Hr51 RNAi expression was limited to adult flies using Gal80^ts^ (*tubGal80*^*ts*^*/+;{Hr51-RNAi}attP2/R14H06-Gal4*), MB morphology was normal compared to controls (*tubGal80*^*ts*^*/+;{mCherry-RNAi}attP2/R14H06-Gal4*), as revealed by anti-FasII labeling. Arrows indicate where MBγ lobes should be. Scale bars represent 50 microns. Temperature shift protocols for Gal80^ts^ experiments are shown (right). (**G**) Venn diagram (Left) showing the overlap of genes that were found to be down-regulated in the MB compared to genetic background controls. RNA-seq was performed on MB nuclei that were isolated using INTACT from *trx*^*RNAi*^ MBs (*UAS-unc84*::*GFP/{trx-RNAi-2}v60000;R14H06-Gal4/UAS-Dcr2*) and genetic background control MBs (*UAS-unc84*::*GFP/v60000;R14H06-Gal4/UAS-Dcr2*) as well as *Set1*^*RNAi*^ (*UAS-unc84*::*GFP/+; R14H06-Gal4/{Set1-RNAi-1}attP2*) and *trr*^*RNAi*^ (*UAS-unc84*::*GFP/+;R14H06-Gal4/{trr-RNAi-3}attP2*) MBs and their genetic background controls (*UAS-unc84*::*GFP/+;R14H06-Gal4/attP2*). A bar graph (right) indicates the percent of unique down-regulated genes identified in *trx*^*RNAi*^, *trr*^*RNAi*^, and *Set1*^*RNAi*^ MBs. (**H**) Heatmap showing the overlap statistics for genes that were down-regulated in *trx*^*RNAi*^, *trr*^*RNAi*^, and *Set1*^*RNAi*^ MBs. Color indicates Jaccard Index. *P*-values are indicated (Fisher’s exact test). (**I, J**) Dot plots showing the normalized counts from INTACT RNA-seq in *Set1*^*RNAi*^ (**I**) and *trr*^*RNAi*^ (**J**) MBs and control MBs for *Ldh*, *Hr51*, *Dgp-1*, and *MFS3*. Statistical significance was determined using a Wald test.(TIF)

S5 Fig Related to Fig 7. **(A)** Dot plots showing FRET ratio in from MBγ (left panel) or MBα (right panel) lobes following a 20-min treatment with 0 mM or 40 mM L-lactate dissolved in PBS. Statistical significance was determined using a Student’s *t* test. Adjacent sample images show differences in FRET ratio corresponding to the 0 mM lactate (upper panel) and 40 mM lactate (bottom panel). Scale bars indicate 50 microns. Genotype: *UAS-laconic/+;R14H06-Gal4/+*. **(B)** Dot plots showing laconic (left panel) or pyronic (right panel) FRET ratio in MBα lobes of flies with MB-specific *trx*^*RNAi*^, *Ldh*^*RNAi*^, and genetic background controls. **(C)** Dot plots showing laconic FRET ratios in MBγ lobes of flies with MB-specific *MFS3*^*RNAi*^ and genetic background controls. The adjacent representative confocal slices show FRET ratio in control (upper panel), and *MFS3*^*RNAi*^ (lower panel) brains. Scale bars indicate 50 microns. **(D, E)** Courtship Indices (CIs–dot plots) and memory indices (MIs–bar graphs). The mean and SEM are indicated in dot plots. Full genotypes are indicated. Genetic background controls are represented using grays, MB-specific RNAi knockdown genotypes are indicated using colors. *P*-values for comparison of naïve (N) and trained (T) groups were calculated using the Mann–Whitney test. *P*-values comparing MIs between control and knockdown genotypes were calculated using a randomization test with 10,000 bootstrap replicates. **(D)** Courtship LTM data underlying relative MIs shown in **Fig 7C**. Genes assessed include *GlyP*, *PyK*, *Pdp*, and *Cs1*. **(E)** Courtship STM of metabolic genes in **Fig 7C** shown to have a LTM phenotype. All raw data associated with this figure are available in **S1 Data.** *n*.*s*. not significant, *p < 0.05, **p < 0.01, ***p < 0.001.
(TIF)

S1 Table Differential gene expression in *trx*^*RNAi*^ MBs compared to controls. (XLSX)

S2 Table Differential H3K4me1 peaks in *trx*^*RNAi*^ MBs compared to control MBs. (XLSX)

S3 Table Differential H3K4me3 peaks in *trx* ^*RNAi*^ MBs compared to control MBs. (XLSX)

S4 Table Differential expression of Trx target genes in the MB compared to WH. (XLSX)

S5 Table Crosses and genotypes. (XLSX)

S1 Data Raw data. (XLSX)

## Abbreviations

ANL: azidonorleucine
ANLS: astrocyte-neuron lactate shuttle
BAC: bacterial artificial chromosome
CI: courtship index
DSHB: Developmental Studies Hybridoma Bank
FRiP: fraction of reads in peaks
O: Gene Ontology
INTACT: isolation of nuclei in a tagged cell type
LTM: long-term memory
MB: mushroom body
MI: memory index
NDD: neurodevelopmental disorder
STM: short-term memory
TF: transcription factor
TIG: training-induced gene
WH: whole head
