# Supplementary figures and images for: Trithorax regulates long-term memory in Drosophila through epigenetic maintenance of mushroom body metabolic state and translation capacity
Source: PLoS Biol. 2025 Jan 27;23(1):e3003004. doi: 10.1371/journal.pbio.3003004 (PMC11835295; doi:10.1371/journal.pbio.3003004)

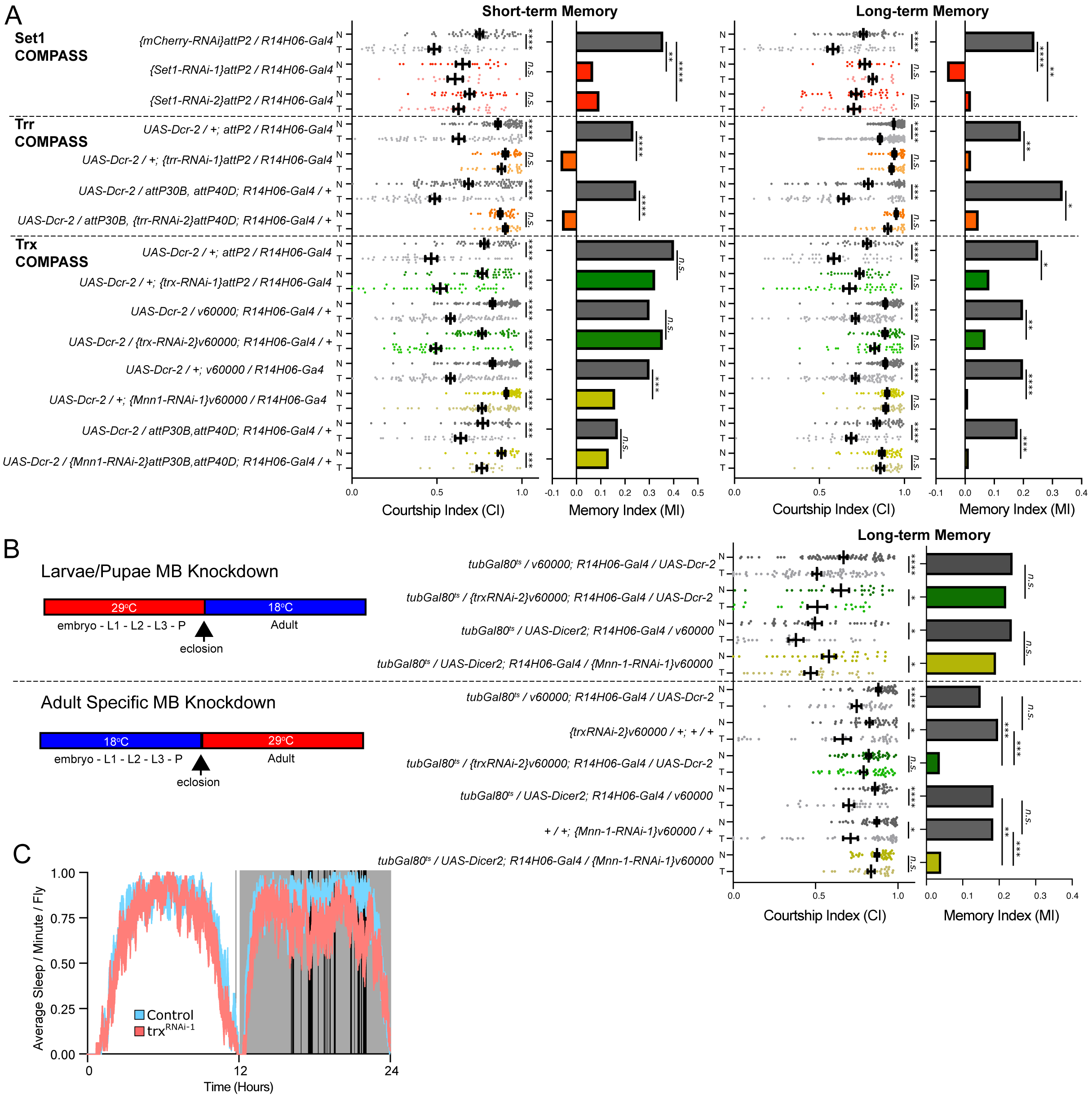

Supplement: S1 Fig — (A, B) Courtship indices (CIs–dot plots) and memory indices (MIs—bar graphs) underlying relative MIs shown in Fig 1A and 1B. The mean and SEM are indicated in dot plots. Full genotypes are indicated. Genetic background controls are represented using grays, MB-specific RNAi knockdown genotypes are indicated using colors. P-values comparing naive (N) and trained (T) flies were generated using a Mann–Whitney test. MIs are calculated from CIs using the formula: MI = (x¯ CInaive - x¯ CItrained) / x¯ CInaive. Statistical significance between MIs was determined using a randomization test with 10,000 bootstrap replicates. (A) Courtship STM (left panel) and LTM (right panel) was assessed upon knockdown of Drosophila COMPASS subunits, Set1, trr, trx, and Mnn1. Previously published data for Set1 [27] is shown for comparison. (B) Gal80ts was used to restrict MB specific trxRNAi and Mnn1RNAi to the larvae/pupae stage or the adult stage. For larvae/pupae knockdown, MB RNAi flies and genetic controls were raised at 29°C and transferred to 18°C at eclosion to prevent RNAi expression in adults. For adult-specific knockdown, flies were raised at 18°C and shifted to 29°C at eclosion. (C) Ribbon plot showing average sleep per minute per fly of MB specific trxRNAi flies (red: UAS-Dcr2/{trx-RNAi-2}v60000; R14H06-Gal4/+) compared to controls (blue: UAS-Dcr2/v60000;R14H06-Gal4/+), averaged over 48 h. Flies are considered asleep if they exhibit no activity over at least 5 min. Average sleep is calculated as the average of sleeping flies (value: 1) or awake flies (value: 0). Line thickness is mean sleep, +/- SEM. White and gray backgrounds indicate objective day and night, respectively. Vertical black lines indicate at least 2 contiguous blocks of statistically significant differences in sleep behavior, measured using Student’s t test. Raw data associated with this figure are available in S1 Data. (TIF) [file pbio.3003004.s001.tif]

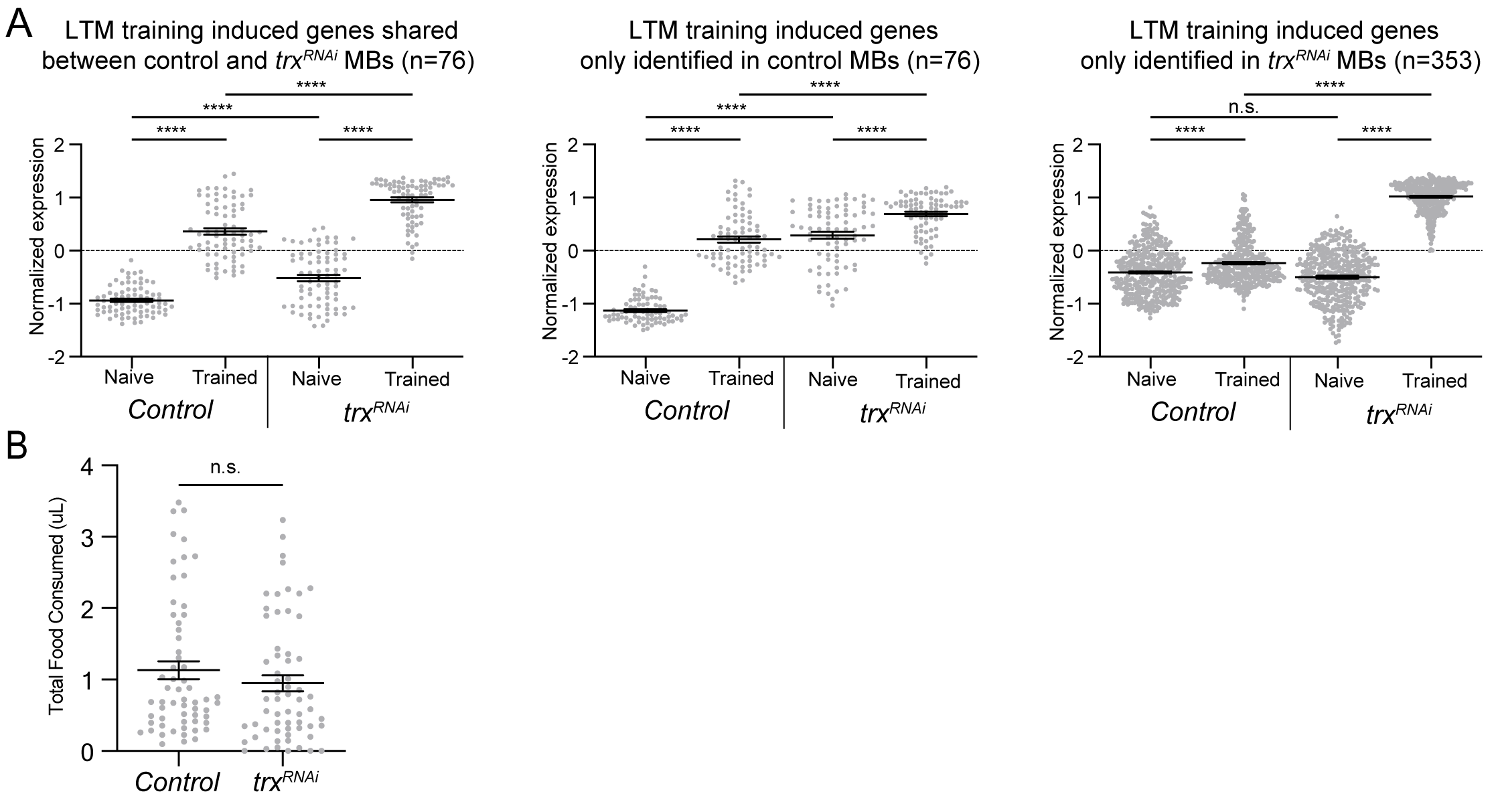

Supplement: S2 Fig — (A) Dot plots showing normalized expression values for: (1) training 0-induced genes identified in both control and trxRNAi MBs (left panel), (2) training-induced genes identified only in control MBs (middle), and (3) training-induced genes identified only in trxRNAi MBs (right). Control genotype: UAS-unc84::GFP/v60000;R14H06-Gal4/UAS-Dcr2. trxRNAi genotype: UAS-unc84::GFP/{trx-RNAi-2}v60000;R14H06-Gal4/UAS-Dcr2. P-values were calculated using pairwise Wilcoxon tests. ****p < 0.0001. (B) Dot plot showing food consumed by trxRNAi flies (red: UAS-Dcr2/{trx-RNAi-2}v60000;R14H06-Gal4/+) compared to controls (blue: UAS-Dcr2/v60000;R14H06-Gal4/+) over a 24-h period. Statistical significance was determined using Student’s t test. n.s.–not significant. Raw data associated with this figure are available in S1 Data. (TIF) [file pbio.3003004.s002.tif]

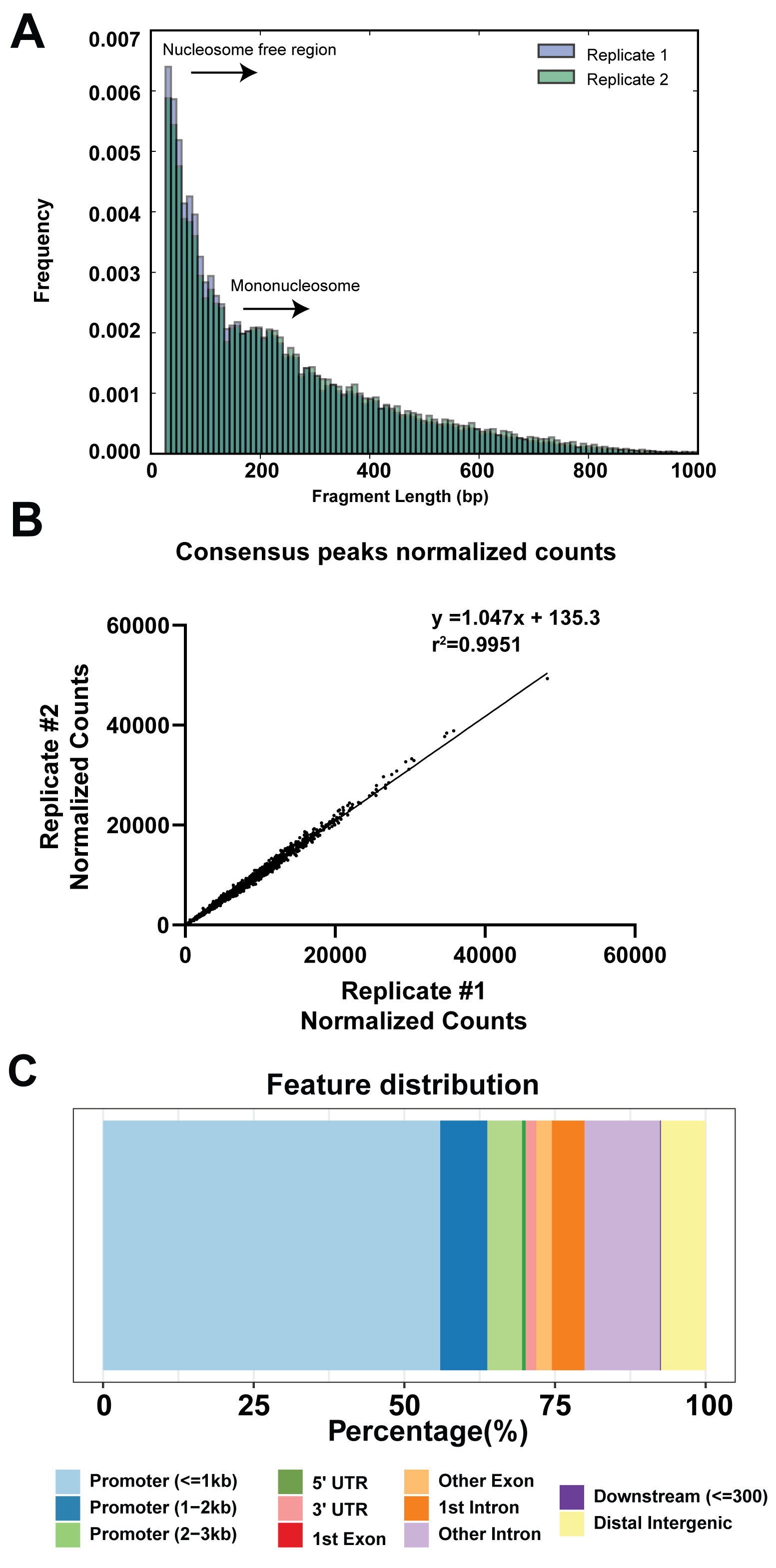

Supplement: S3 Fig — (A) Fragment size distribution of ATAC-seq libraries generated from INTACT-isolated MB nuclei. Distribution for 2 biological replicates is shown. Peak signal of nucleosome free regions (80–120 bp) and mononucleosomes (~180 bp) is marked. (B) Scatter plot of normalized counts for consensus peaks from MB ATAC-seq samples. Each dot represents a consensus peak, with positions indicating normalized counts in replicate 1 and replicate 2. Line of best fit is shown, with corresponding linear equation, and coefficient of determination. (C) Feature distribution of annotated ATAC-seq peaks, which are predominantly located near transcriptional start sites (TSS) of genes. (TIF) [file pbio.3003004.s003.tif]

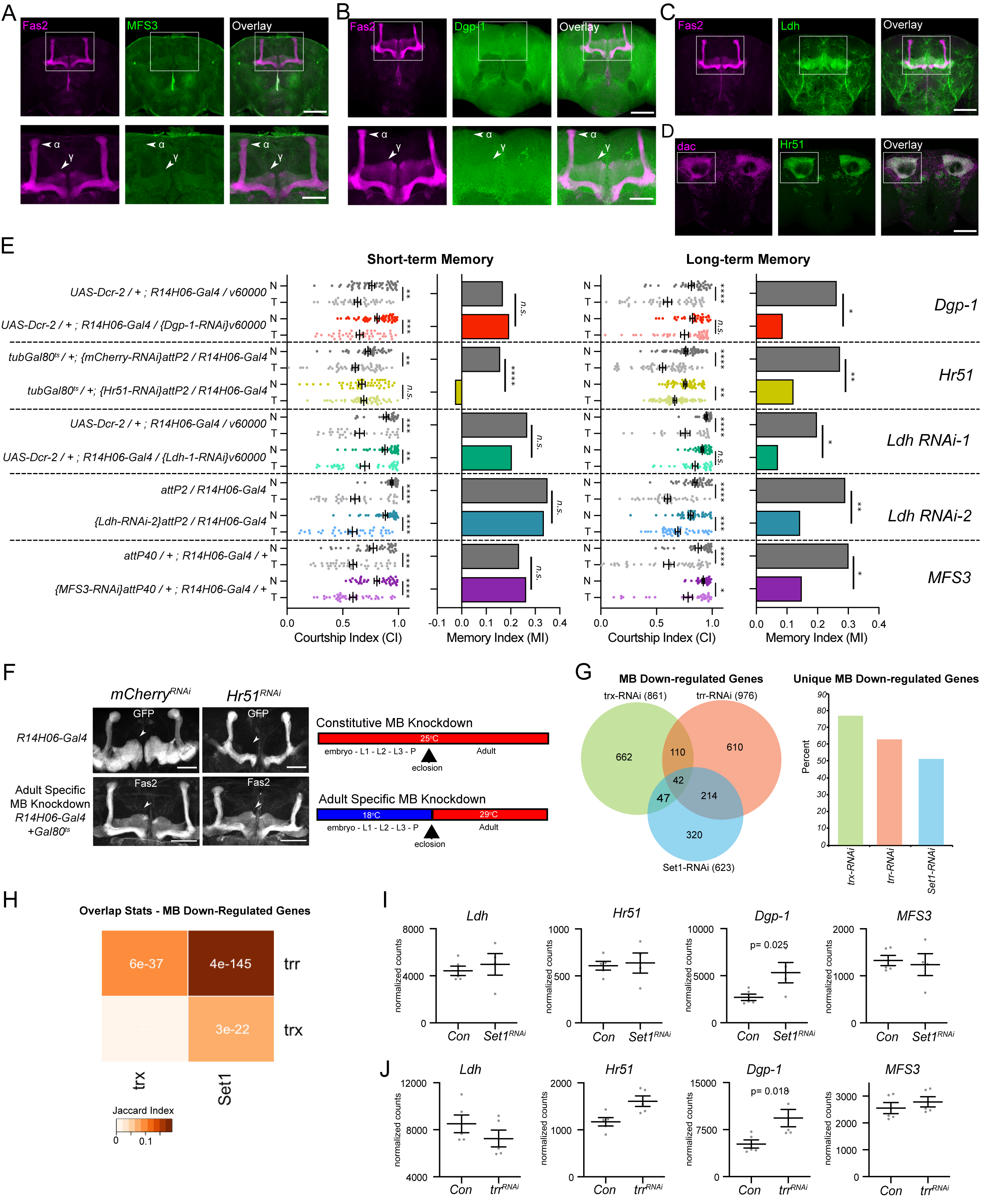

Supplement: S4 Fig — (A–D) Confocal z-stack projections showing localization of (A) MFS3::YFP, (B) Dgp-1::GFP, (C) Ldh::GFP, and (D) Hr51::GFP (middle panels) in the (A–C) MB lobes (anti-Fas2 –left panel) or (D) MB nuclei (anti-dac–left panel). In (A) and (B) regions of interest are defined by a white box and shown immediately below. For (C) and (D) regions of interesting are defined by a white box and shown in Fig 4C and 4D. Scale bars indicate 50 microns. (E) Courtship indices (CIs–dot plots) and memory indices (MIs-bar graphs) underlying relative MIs from Fig 4E. The mean and SEM are indicated in dot plots. Full genotypes are shown. Genetic background controls are represented using grays, MB-specific RNAi knockdown genotypes are indicated using colors. P-values comparing naive (N) and trained (T) flies were generated using a Mann–Whitney test. MIs were calculated from CIs using the formula: MI = (x¯ CInaive - x¯ CItrained) / x¯ CInaive). Statistical significance between MIs was determined using a randomization test with 10,000 bootstrap replicates. Raw data associated with this figure are available in S1 Data. (F) Confocal z-stack projections showing the impact of Hr51RNAi on MB morphology. Unrestricted knockdown (i.e., without Gal80ts) of Hr51 in the MB with R14H06-Gal4 at 25°C (UAS-MetRS*::GFP/+;{Hr51-RNAi}attP2/R14H06-Gal4) led to loss of MBγ lobes that was not observed in controls (UAS-MetRS*::GFP/+;{mcherry-RNAi}attP2/R14H06-Gal4). When Hr51 RNAi expression was limited to adult flies using Gal80ts (tubGal80ts/+;{Hr51-RNAi}attP2/R14H06-Gal4), MB morphology was normal compared to controls (tubGal80ts/+;{mCherry-RNAi}attP2/R14H06-Gal4), as revealed by anti-FasII labeling. Arrows indicate where MBγ lobes should be. Scale bars represent 50 microns. Temperature shift protocols for Gal80ts experiments are shown (right). (G) Venn diagram (Left) showing the overlap of genes that were found to be down-regulated in the MB compared to genetic background controls. RNA-seq was performed on MB [file pbio.3003004.s004.tif]

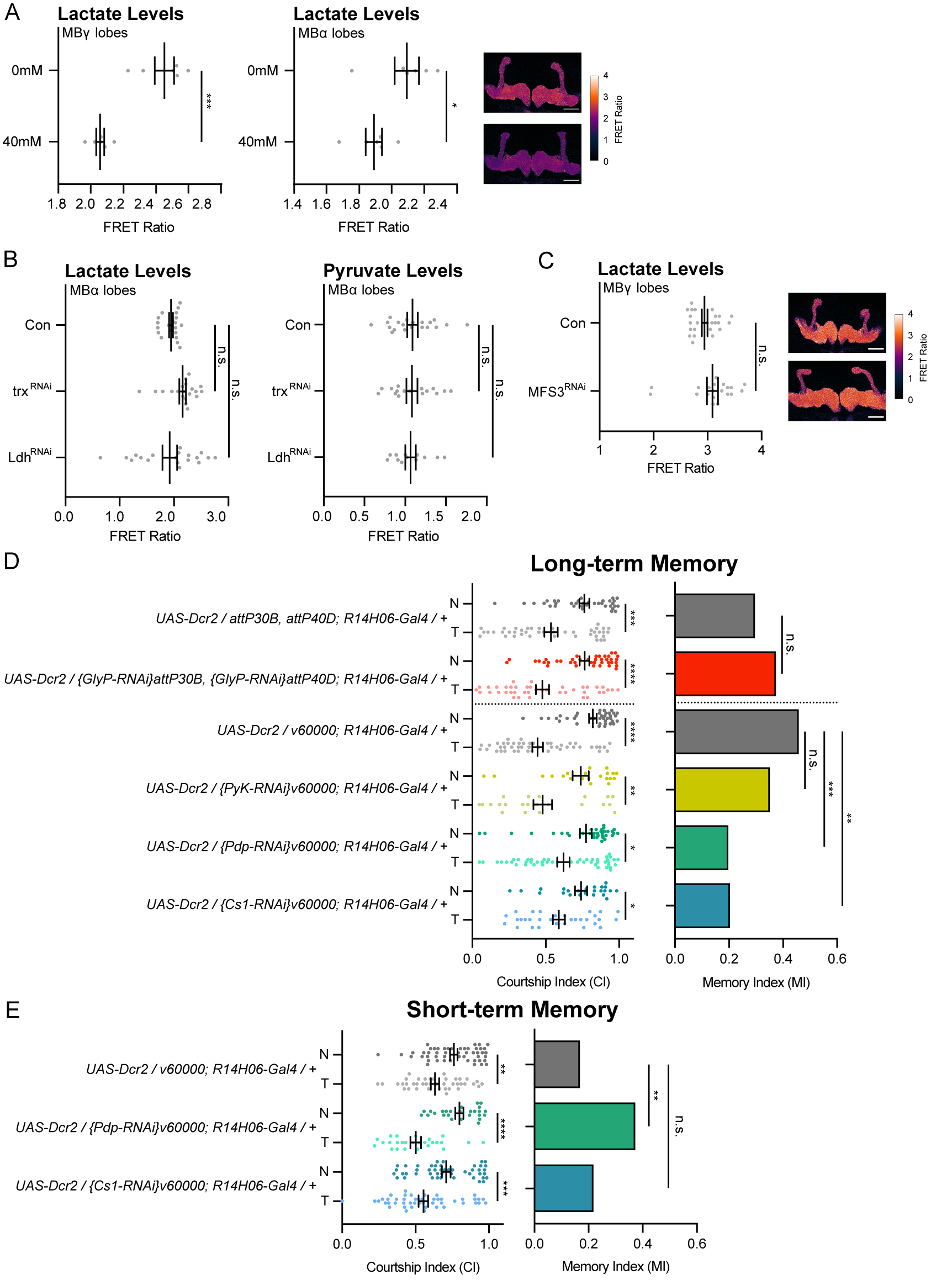

Supplement: S5 Fig — (A) Dot plots showing FRET ratio in from MBγ (left panel) or MBα (right panel) lobes following a 20-min treatment with 0 mM or 40 mM L-lactate dissolved in PBS. Statistical significance was determined using a Student’s t test. Adjacent sample images show differences in FRET ratio corresponding to the 0 mM lactate (upper panel) and 40 mM lactate (bottom panel). Scale bars indicate 50 microns. Genotype: UAS-laconic/+;R14H06-Gal4/+. (B) Dot plots showing laconic (left panel) or pyronic (right panel) FRET ratio in MBα lobes of flies with MB-specific trxRNAi, LdhRNAi, and genetic background controls. (C) Dot plots showing laconic FRET ratios in MBγ lobes of flies with MB-specific MFS3RNAi and genetic background controls. The adjacent representative confocal slices show FRET ratio in control (upper panel), and MFS3RNAi (lower panel) brains. Scale bars indicate 50 microns. (D, E) Courtship Indices (CIs–dot plots) and memory indices (MIs–bar graphs). The mean and SEM are indicated in dot plots. Full genotypes are indicated. Genetic background controls are represented using grays, MB-specific RNAi knockdown genotypes are indicated using colors. P-values for comparison of naïve (N) and trained (T) groups were calculated using the Mann–Whitney test. P-values comparing MIs between control and knockdown genotypes were calculated using a randomization test with 10,000 bootstrap replicates. (D) Courtship LTM data underlying relative MIs shown in Fig 7C. Genes assessed include GlyP, PyK, Pdp, and Cs1. (E) Courtship STM of metabolic genes in Fig 7C shown to have a LTM phenotype. All raw data associated with this figure are available in S1 Data. n.s. not significant, *p < 0.05, **p < 0.01, ***p < 0.001. (TIF) [file pbio.3003004.s005.tif]
